# Comprehensive In Vitro, Genomic, Microbiota, and Subacute Toxicological Safety Characterization of *Lactiplantibacillus plantarum* ATA-LPC98052

**DOI:** 10.3390/nu18172796

**Published:** 2026-08-26

**Authors:** Şükran Özdatlı Kurtuluş, Ertuğrul Osman Bursalıoğlu, Reyhan Aliusta, Betül Türker Şallı, Batuhan Cenk Özkan, Deniz Köşebent, Ahmet Arif Kurt, Abdurrahman Recep Bülbül, Fatih Hacımustafaoğlu, Bekir Çakıcı, Nazlı Gamze Bülbül, Emine Kızılay, Ronayı Coşkun, Gamze Yıldırım, Semra Sardas, İsmail Aslan

**Affiliations:** 1 Department of Pharmaceutical Toxicology, Hamidiye Faculty of Pharmacy, University of Health Sciences, Istanbul 34668, Türkiye; sukranozdatli.kurtulus@sbu.edu.tr; 2 Department of Medical Services and Techniques, Vocational School of Health Services, Sinop University, Sinop 57000, Türkiye; ebursalioglu@sinop.edu.tr; 3 Venar Sağlık A. S., Büyükesat Mah., Çayhane St., No: 35/A GOP, Çankaya, Ankara 06700, Türkiye; reyhaneser@hotmail.com; 4 ATABIO Technologies Co., Ltd., Teknopol Istanbul, Istanbul 34903, Türkiye; betul.turker@sfaarge.com (B.T.Ş.); batuhan.ozkan@atabio.com.tr (B.C.Ö.); deniz.kosebent@atabio.com.tr (D.K.); rrecepp3737@gmail.com (A.R.B.); bekir.cakici@sfaarge.com (B.Ç.); 5 SFA R&D Private Health Services Co., Ltd., Teknopark Istanbul, Istanbul 34890, Türkiye; 6 Department of Pharmaceutical Technology, Faculty of Pharmacy, Süleyman Demirel University, Isparta 32000, Türkiye; ahmetkurt@sdu.edu.tr; 7 Medical Laboratory Techniques Program, Department of Medical Services and Techniques, Hamidiye Vocational School of Health Services, University of Health Sciences, Istanbul 34668, Türkiye; fatih.hacimustafaoglu@sbu.edu.tr (F.H.); emine.kizilay@sbu.edu.tr (E.K.); 8 Department of Neurology, Faculty of Medicine, University of Health Sciences, Istanbul 34668, Türkiye; nzl.gmzb@gmail.com; 9 Department of Pharmacy Management, Hamidiye Faculty of Pharmacy, University of Health Sciences, Istanbul 34668, Türkiye; ronayi.coskun@sbu.edu.tr; 10 Kızılay Beverages, Istanbul 34750, Türkiye; gamzey100@gmail.com; 11 Department of Pharmaceutical Sciences, Faculty of Pharmacy, Istinye University, Istanbul 34408, Türkiye; semrasardas@gmail.com; 12 Department of Pharmaceutical Technology, Hamidiye Faculty of Pharmacy, University of Health Sciences, Istanbul 34668, Türkiye

**Keywords:** *Lactiplantibacillus plantarum*, whole-genome sequencing, subacute oral toxicity, gut microbiota

## Abstract

Background: *Lactiplantibacillus plantarum* is a widely studied probiotic species; however, probiotic characteristics and safety profiles are strain-specific, requiring independent evaluation. This study characterized *Lactiplantibacillus plantarum* ATA-LPC98052 as a candidate probiotic raw material. Methods: ATA-LPC98052 was evaluated for hemolysis, acid/bile tolerance, Caco-2 adhesion, cytotoxicity, and storage stability. Subacute oral safety was assessed in Wistar rats by gavage for 28 days at 1.2 × 10^11^ CFU/kg/day; the study design incorporated selected principles of OECD Test Guideline 407. Clinical, hematological, biochemical, organ-weight, and macroscopic endpoints were evaluated. Fecal microbiota was analyzed by 16S rRNA sequencing; WGS was used for taxonomic confirmation and genomic safety screening. Results: ATA-LPC98052 was γ-hemolytic and maintained 60% viability at pH 1.5 and 96% at pH 5.0, while viability ranged from 74% to 82% across 0.1–0.5% bile salt concentrations. Caco-2 cell viability was 99%, adhesion exceeded 90%, and the lyophilized preparation remained stable for 15 months, maintaining 9.6 log_10_ CFU/g. Repeated oral administration caused no mortality or consistent treatment-related toxicological pattern. Longitudinal microbiota analysis showed no significant treatment × time effects on alpha diversity or Bray–Curtis community structure, and no genus-level MaAsLin2 association was FDR-significant. WGS confirmed *L. plantarum* identity and no contamination; ResFinder detected no acquired antimicrobial resistance determinants meeting specified thresholds, whereas CARD/RGI identified low-identity *qacJ* and *vanY* homologs requiring cautious interpretation. Conclusion: ATA-LPC98052 demonstrated favorable in vitro probiotic characteristics, technological stability, gut microbiota-modulating potential, and a favorable subacute safety profile under the tested conditions; however, microbiota findings were exploratory, and phenotypic MIC testing remains warranted.

## 1. Introduction

*Lactiplantibacillus plantarum* (formerly *Lactobacillus plantarum*) is a Gram-positive, rod-shaped, non-spore-forming lactic acid bacterium that exhibits facultative heterofermentative metabolism. The species displays a broad ecological niche distribution, spanning dairy products, fermented vegetables, the human gastrointestinal tract and vaginal microbiota, as well as fruits, meat, fish, and various traditional fermented foods [1,2]. This widespread distribution is generally attributed to the relatively large genome of approximately 2.8–3.3 Mb and the genomic plasticity (nomadic lifestyle) that enables rapid adaptation to diverse habitats [3,4]; comparative genomic analyses have suggested that genetic diversity among *L. plantarum* strains is concentrated in gene regions associated with exopolysaccharide biosynthesis and carbohydrate metabolism, while ecological specialization remains limited [1].

The species appears to be prominent in probiotic research, which may be attributed to its long history of utilization in fermented foods. Although certain strains and preparations have been evaluated under the GRAS and QPS frameworks, this status should not be interpreted as direct evidence of safety applicable to all strains of the species; independent characterization of each strain remains essential [5,6].

Existing literature has suggested that different *L. plantarum* strains may possess the potential for the modulation of the gut microbiota, the regulation of immune responses, the reduction of oxidative stress, the improvement of metabolic parameters, and the inhibition of undesirable microorganisms through plantaricin-like bacteriocins [7,8,9,10]. However, these effects cannot be assumed to be common across all *L. plantarum* strains. Strains such as 299v, PS128, EGCG 13110402, and HAC01, which belong to the same species, have been investigated in different functional contexts, demonstrating that substantial biological diversity may exist among strains of a single species [10]. Recent advancements in microbiome research have demonstrated that probiotics can effectively normalize local microbiota environments and suppress targeted pathogens outside the gastrointestinal tract, including their successful integration into natural, aluminum-free antiperspirant formulations [11].

Traditional fermented products constitute important natural reservoirs for the discovery of novel probiotic candidates. *L. plantarum* strains isolated from diverse matrices such as Jiangshui, Boza, Chinese fermented foods, Algerian fruits, and human milk have been reported to display marked variability in probiotic-relevant properties including acid and bile tolerance at pH 1.5–3.5, cell surface hydrophobicity, auto-aggregation, antimicrobial activity, and adhesion capacity to Caco-2 cells [7,12,13,14]. This variability may underscore the potential necessity of strain-specific in vitro probiotic characterization for each new isolate.

In addition to in vitro characterization, the preclinical safety of novel probiotic candidates should be supported by repeated-dose oral toxicity studies conducted in accordance with the Organization for Economic Co-operation and Development (OECD) guidelines. Such studies provide fundamental safety data regarding mortality, clinical observations, body weight, feed intake, hematological and biochemical parameters, organ weights, and histopathological findings. Although previous studies with different *L. plantarum* strains have generally reported favorable safety profiles, these data might not be directly extrapolated to newly isolated strains [5,8,15].

ATA-LPC98052 is a soil-derived strain originally isolated from the Spil Mountain region of Manisa, Türkiye. Environmental isolates may possess strain-specific adaptive characteristics; however, their potential probiotic relevance must be established experimentally through gastrointestinal stress tolerance, epithelial interaction, genomic safety screening, and in vivo evaluation. In the present study, the newly isolated *L. plantarum* ATA-LPC98052 strain was evaluated as a candidate probiotic raw material through assessment of hemolytic activity, cytotoxicity, gastric acid and bile tolerance, Caco-2 adhesion capacity, antimicrobial activity, storage stability, whole-genome sequencing–based safety characterization, and a 28-day repeated-dose oral toxicity study. Whole-genome sequencing was performed to confirm strain identity and to screen for acquired antimicrobial resistance genes, while fecal microbiota analysis was conducted to provide supportive evidence of administration-related compositional changes. The findings may provide a preclinical safety and probiotic characterization of ATA-LPC98052 and might establish a foundation for future functional validation studies, including standardized phenotypic antimicrobial susceptibility testing and efficacy evaluation. The potential relevance of ATA-LPC98052 to gut health was evaluated on the basis of its resistance to gastric acidity and bile salts, adhesion to intestinal epithelial cells, and its effects on fecal microbial diversity and taxonomic composition.

## 2. Materials and Methods

### 2.1. Origin and Preparation of the Probiotic Material

*Lactiplantibacillus plantarum* ATA-LPC98052 was originally isolated from a soil sample collected from the Spil Mountain region of Manisa, Türkiye. The strain is maintained in the ATA-LTC Culture Collection under catalogue number 134-P2. Its taxonomic identity was subsequently confirmed by whole-genome sequencing.

*Lactiplantibacillus plantarum* ATA-LPC98052 was produced as a viable lyophilized probiotic preparation under controlled manufacturing conditions. The master culture was activated under anaerobic conditions and propagated in an appropriate culture medium. After reaching the target cell density, the culture was transferred to a bioreactor using a 5% inoculum and anaerobically fermented for 72 h. Following fermentation, bacterial cells were coated with a sodium alginate–inulin formulation, harvested by centrifugation, rapidly frozen at −80 °C, and lyophilized. The sodium alginate–inulin matrix was used as a protective formulation to improve resistance to processing and storage-related stress. The final preparation was standardized to contain approximately 10^9^–10^11^ CFU/g culturable bacteria and stored at controlled room temperature until use [16].

### 2.2. In Vitro Characterization

#### 2.2.1. Gastric Acid and Bile Salt Tolerance

Resistance to simulated gastric acid and bile salts was evaluated under in vitro conditions. For acid tolerance, bacterial suspensions were incubated in phosphate-buffered saline (PBS) adjusted to pH 1.5, 2.0, 3.0, 4.0, and 5.0 for 2 h [17,18], simulating transient gastric stress conditions. Bile salt tolerance was assessed by incubating the strain in brain heart infusion (BHI) broth supplemented with 0.1–0.5% bile salts, indicating a potential for physiological resilience. Following incubation, viable bacteria were quantified by serial dilution and anaerobic plate counting on BHI agar. Results were expressed as CFU/mL.

#### 2.2.2. Caco-2 Cell Culture

Caco-2 cell line (ATCC^®^ HTB-37™, Manassas, VA, USA) was cultured in Dulbecco’s Modified Eagle Medium (DMEM; Gibco, Thermo Fisher Scientific, Waltham, MA, USA) supplemented with 10% fetal bovine serum(Gibco, Thermo Fisher Scientific, Waltham, MA, USA), 1% penicillin–streptomycin (Gibco, Thermo Fisher Scientific, Waltham, MA, USA), and 1% L-glutamine(Gibco, Thermo Fisher Scientific, Waltham, MA, USA); and maintained at 37 °C in a humidified atmosphere containing 5% CO_2_ until the required confluence was reached.

#### 2.2.3. Adhesion Assay and Cytotoxicity

The adhesion ability and cytotoxicity of *L. plantarum* ATA-LPC98052 were evaluated using Caco-2 cells.

For the cytotoxicity assay, cells were seeded into 96-well plates and incubated for 24 h before exposure to the bacterial suspension (2 × 10^6^ CFU/mL). Cell viability was determined using the MTT (Sigma-Aldrich, St. Louis, MO, USA) assay according to ISO 10993-5. Following incubation, MTT reagent was added and formazan crystals were dissolved in DMSO (Sigma-Aldrich, St. Louis, MO, USA). Absorbance was measured at 570 nm, and cell viability was expressed relative to the negative control [19].

For the adhesion assay, confluent Caco-2 monolayers were incubated with the bacterial suspension for 24 h. Non-adherent bacteria were removed by washing with sterile PBS, whereas adherent bacteria were recovered by cell lysis and quantified by plate counting. Adhesion was expressed as the percentage of the initial inoculum.

#### 2.2.4. Hemolytic Activity

Hemolytic activity was evaluated by streaking *L. plantarum* ATA-LPC98052 onto sheep blood agar plates in 10 independent replicates. Plates were incubated anaerobically at 37 °C for 48–72 h and subsequently examined for the presence of hemolytic zones. Hemolysis may be classified as α-, β-, or γ-hemolysis, contingent upon the phenotypic appearance of the surrounding agar.

#### 2.2.5. Long-Term Stability

The stability of the lyophilized preparation was assessed during storage at controlled room temperature for 15 months. Samples were analyzed at baseline and at monthly intervals. At each time point, the preparation was reconstituted, serially diluted, and plated on BHI agar under anaerobic conditions. BHI agar was selected as a nutrient-rich, non-selective recovery medium to support the recovery and culturability of ATA-LPC98052 cells potentially subjected to sublethal injury during lyophilization and prolonged storage, thereby reducing the potential underestimation of culturable cells. Culturable bacterial counts were determined after incubation and expressed as log_10_ CFU/g [20]. Representative microscopic images illustrating the cellular morphology of *Lactiplantibacillus plantarum* ATA-LPC98052 at increasing magnifications are provided in Supplementary Figure S1.

### 2.3. Animal Study

#### 2.3.1. Ethical Approval and Animal Husbandry

The animal study was reported in accordance with the ARRIVE 2.0 guidelines. The 28-day repeated-dose oral study design was adapted from the general principles of OECD Test Guideline 407 to provide supplementary preclinical safety information for the newly characterized strain; it was not intended as a formal regulatory OECD compliance study. The experimental protocol was approved by the IBG Local Animal Experimentation Ethics Committee (Approval No. 2025-011; approved 10 April 2025). Determination of group size adhered to OECD Guideline 407, which recommends five animals of each sex per experimental cohort. Specific pathogen-free Wistar albino rats (six to eight weeks of age; 180–220 g) were obtained from a certified breeding facility and underwent a five-day acclimatization period prior to study initiation.

Animals were housed individually with one animal per conventional cage providing a minimum floor area of 800 cm^2^, under controlled environmental conditions (22 ± 3 °C, 50 ± 20% relative humidity, 12 h light/12 h dark cycle). Standard laboratory rodent chow and drinking water were provided *ad libitum*, and environmental enrichment was supplied throughout the study.

#### 2.3.2. Experimental Design

A 28-day repeated-dose oral study, adapted from selected principles of OECD Test Guideline 407, was conducted to obtain supplementary safety information following repeated administration of ATA-LPC98052. The administered dose (1000 mg/kg/day; approximately 1.2 × 10^11^ CFU/kg/day) was selected on the basis of prior safety investigations, in which oral administration of approximately 4000 mg/kg/day for three weeks produced no treatment-related toxicity [16].

Animals were randomly assigned to negative control (NC) and *Lactiplantibacillus plantarum* ATA-LPC98052 treatment groups, each consisting of five males and five females. Formal blinding was not implemented during the study. However, all groups were handled by the same personnel under the same environmental conditions, dosing schedule, and equivalent experimental procedures. The probiotic preparation or vehicle (drinking water) was administered once daily by oral gavage for 28 consecutive days. Dose volume was adjusted weekly according to individual body weight measurements. At the end of the treatment period, animals were euthanized for toxicological evaluation. Additional recovery groups, each consisting of five female and five male rats, were maintained for a 14-day treatment-free recovery period to assess the reversibility or persistence of any treatment-related effects.

#### 2.3.3. Clinical Observations and Functional Assessment

The examination of all animals was performed prior to dosing, while monitoring for mortality and morbidity was conducted twice daily throughout the investigation. Detailed clinical observations, which may encompass assessments of general appearance, cutaneous integrity, ocular and mucous membrane status, physiological system functionality, and behavioral patterns, were subsequently performed.

Functional observations were performed during Week 4 in accordance with OECD Guideline 407 and included sensory reactivity, grip strength, and spontaneous motor activity. Body weights were recorded before the first dose, weekly throughout the treatment period, and prior to necropsy. Food consumption was measured weekly.

### 2.4. Sample Collection and Toxicological Evaluation

#### 2.4.1. Blood Collection and Hematological/Biochemical Analyses

At the end of the 28-day treatment period and after the 14-day recovery period, animals were fasted overnight while water remained available ad libitum. Deep anesthesia was induced with inhaled isoflurane, and anesthetic depth was confirmed by the absence of the pedal withdrawal reflex in response to a firm toe pinch. While the animals remained under deep anesthesia, blood was collected by cardiac puncture. Euthanasia was completed by exsanguination followed by cervical dislocation before necropsy. Blood intended for hematological analyses was collected into anticoagulant-treated tubes, whereas samples for serum biochemistry were collected into separator gel tubes and processed according to standard laboratory procedures.

Hematological parameters, including white blood cell count (WBC), lymphocytes (LYM and LYM%), monocytes (MON and MON%), granulocytes (GRA and GRA%), red blood cell count (RBC), hemoglobin (HGB), hematocrit (HCT), and platelet count (PLT), were determined using an automated hematology analyzer (Boule Exigo H400, Boule Medical AB, Stockholm, Sweden), a methodology which may provide precise quantification of cellular indices.

Serum biochemical analyses included alanine aminotransferase (ALT), aspartate aminotransferase (AST), alkaline phosphatase (ALP), urea, creatinine, glucose, total cholesterol, triglycerides, albumin, total protein, total bilirubin, sodium, potassium, calcium, *C*-reactive protein (CRP), and clotting time. Measurements were performed using an automated clinical chemistry analyzer (RX Daytona Plus, Randox Laboratories, Antrim, UK) according to the manufacturer’s instructions.

#### 2.4.2. Necropsy and Organ Weight Determination

Following blood collection, all animals underwent complete necropsy. Organs and tissues specified in OECD Guideline 407 were examined macroscopically. Absolute organ weights were recorded for the brain, heart, liver, kidneys, spleen, thymus, adrenal glands, and reproductive organs. Relative organ weights were calculated by normalizing organ weights to terminal body weight, an approach that facilitates a more nuanced assessment of physiological development.

#### 2.4.3. Histopathological Evaluation

Representative tissues were fixed in 10% neutral buffered formalin, routinely processed, embedded in paraffin, sectioned at 4 μm, and stained with hematoxylin and eosin (H&E). Subsequent histopathological examinations were conducted by a board-certified veterinary pathologist utilizing light microscopy.

### 2.5. Gut Microbiota Analysis by 16S rRNA Gene Sequencing

Gut microbiota composition was evaluated by 16S rRNA gene sequencing following approval of the study by the Izmir Biomedicine and Genome Center Ethics Committee (Approval No. 2025-003; approved 10 April 2025). Fecal samples from control and *Lactiplantibacillus plantarum* ATA-LPC98052-treated female Wistar rats were collected at baseline (Day 0) and after the intervention period (Day 15) for longitudinal microbial diversity and taxonomic analyses.

Genomic DNA was extracted from fecal samples using the High Pure PCR Template Preparation Kit (Roche Diagnostics, Mannheim, Germany) according to the manufacturer’s instructions. DNA quality and concentration were assessed using the Quant-iT PicoGreen dsDNA Assay Kit (Thermo Fisher Scientific, Waltham, MA, USA). The V3-V4 hypervariable region of the bacterial 16S rRNA gene (approximately 470 bp) was amplified using the universal primer pair 341F (5′-CCTAYGGGRBGCASCAG-3′) and 806R (5′-GGACTACNNGGGTATCTAAT-3′). Amplicons were indexed using the Nextera XT Index Kit (Illumina, San Diego, CA, USA), pooled at equimolar concentrations, and sequenced on an Illumina MiSeq platform using MiSeq Reagent Kit v3 (Illumina, San Diego, CA, USA) chemistry (2 × 250 bp paired end reads).

Raw sequencing data were processed using QIIME2 (version 2023.2). Sequence denoising, chimera removal, and amplicon sequence variant (ASV) inference were performed using the DADA2 v1.38 pipeline. Taxonomic assignment was carried out against the SILVA reference database (version 138). Only sequences assigned to bacterial taxa were retained for downstream analyses, whereas unassigned and non-bacterial sequences were excluded. Downstream analyses were conducted in R (version 4.3.1) using the phyloseq (version 1.44.0) framework.

Alpha diversity was evaluated using the Shannon, Simpson, inverse Simpson, and Pielou’s evenness indices. To account for repeated measurements within individual animals, longitudinal alpha-diversity analyses were performed using linear mixed-effects models with treatment, time, and the treatment × time interaction as fixed effects and individual rat identity as a random effect. Beta diversity was assessed using Bray–Curtis dissimilarity and visualized by principal coordinates analysis (PCoA). Differences in overall microbial community composition were tested using PERMANOVA, while homogeneity of multivariate dispersion was evaluated using PERMDISP. The overall four-group PERMANOVA used 9999 permutations; for the longitudinal treatment × time assessment, permutations were restricted within individual rat identity to account for the repeated-measures structure.

Differential abundance was evaluated primarily at the genus level using MaAsLin2 v3.23 in a longitudinal model including treatment, time, and the treatment × time interaction. Model coefficients, standard errors, raw *p*-values, and false-discovery-rate-adjusted q-values were reported. A q-value < 0.05 was considered statistically significant after correction for multiple testing, whereas findings at q < 0.25 were considered exploratory. Species-level analysis was performed as a supplementary exploratory analysis because taxonomic assignment at the species level from short 16S rRNA amplicons is less reliable.

### 2.6. Whole-Genome Sequencing and Genomic Safety Assessment

Genomic DNA was extracted from the pure culture of *Lactiplantibacillus plantarum* ATA-LPC98052 using a semi-automated Kurabo Mini-80 system (Kurabo, Osaka, Japan). DNA libraries were prepared using the Nextera XT DNA Library Preparation Kit and sequenced on an Illumina short-read sequencing platform (Illumina, San Diego, CA, USA). Raw FASTQ files were used for downstream bioinformatic analysis. Taxonomic identification was performed using MetaPhlAn4 v4.2.5, facilitating Bowtie2 v2.5.5 mapping against the CHOCOPhlAn database, which suggests a high degree of resolution. Functional gene, protein, enzyme, and metabolic pathway annotations were generated using HUMAnN v3.9 with UniRef90 and MetaCyc databases. De novo genome assembly was performed using SPAdes v3.15 with the “-isolate” option, and assembly quality was assessed using QUAST v5.2.0. The assembled genome was deposited in the NCBI database under BioSample accession SAMN40643420 and BioProject accession PRJNA1092961.

Antimicrobial resistance determinants were screened using the DTU/GROOT v1.1 pipeline integrated with the ResFinder v2.6.0 database. ResFinder screening was performed using minimum thresholds of 80% sequence identity and 60% aligned gene length/coverage. As an additional sensitivity analysis, both the sequence identity and gene coverage thresholds were progressively reduced in parallel to approximately 50%; no additional acquired antimicrobial resistance determinants were identified under these lower-stringency conditions. Additional antimicrobial resistance screening was performed using CARD (v4.0.1). The analysis was restricted to Perfect and Strict hits, with nudge-based reclassification of Loose hits to Strict excluded, and high sequence quality/coverage criteria were applied. Virulence-associated genes were screened using ABRicate v1.2.0 against the VFDB database (database version: 15 March 2026). Screening was performed across combinations of minimum sequence identity and gene coverage thresholds ranging from 50% to 90% in 10% increments to evaluate the persistence of database matches under increasing stringency.

Standardized phenotypic minimum inhibitory concentration (MIC) testing was not included within the experimental scope of the present study. Therefore, the antimicrobial resistance assessment was limited to genomic screening and should not be interpreted as a complete phenotypic antimicrobial susceptibility evaluation.

### 2.7. Statistical Analysis

Statistical analyses were performed separately for female and male rats. For endpoint variables measured once at necropsy (hematological, biochemical, organ weight, and clotting parameters), comparisons between the negative control and *L. plantarum*-treated groups were performed using the unpaired Student’s *t*-test for normally distributed data or the Mann–Whitney *U* test for non-normally distributed data, as these comparisons involved independent experimental groups. Body weight and food consumption were recorded repeatedly throughout the study; however, comparisons were performed between independent treatment groups at each corresponding time point. A two-sided *p* value < 0.05 was considered statistically significant.

The statistical methods used for gut microbiota analysis are described in Section 2.6.

## 3. Results

### 3.1. In Vitro Characterization of Lactiplantibacillus plantarum ATA-LPC98052

#### 3.1.1. Hemolytic Activity Assessment

The hemolytic activity of *Lactiplantibacillus plantarum* ATA-LPC98052 was evaluated on sheep blood agar using 10 independent replicates. As expected, *Clostridium perfringens* and *Staphylococcus aureus* exhibited β- and α-hemolytic activity, respectively. In contrast, no hemolytic zones were observed for ATA-LPC98052 in any replicate, a finding which suggests the presence of a γ-hemolytic phenotype.

#### 3.1.2. Acid and Bile Tolerance

**Figure 1:**
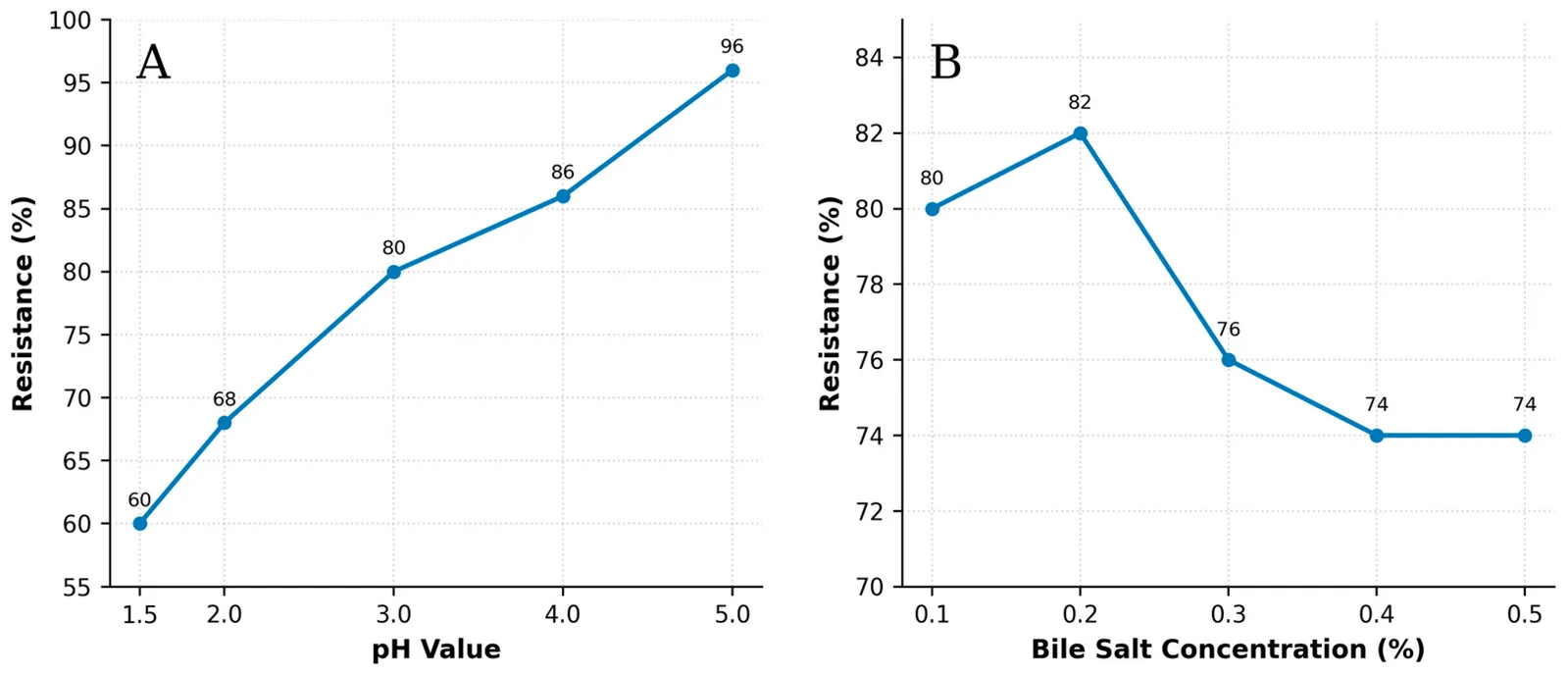
Gastric acid and bile salt tolerance of *Lactiplantibacillus plantarum* ATA-LPC98052. (**A**) Survival after 2 h exposure to acidic conditions (pH 1.5–5.0). (**B**) Viability following exposure to 0.1–0.5% bile salts. Data are expressed as CFU-based survival.

The survival of ATA-LPC98052 under simulated gastrointestinal conditions is presented in Figure 1. After 2 h of incubation, the strain retained 60% viability at pH 1.5, with survival increasing progressively as pH increased, reaching 96% at pH 5.0 (Figure 1A). Under bile salt challenge, viability ranged from 74% to 82% across the tested concentrations of 0.1–0.5%, a result which may indicate high tolerance to physiologically relevant bile salt levels (Figure 1B).

#### 3.1.3. Adhesion Capacity, Cytotoxicity and Long-Term Stability

**Figure 2:**
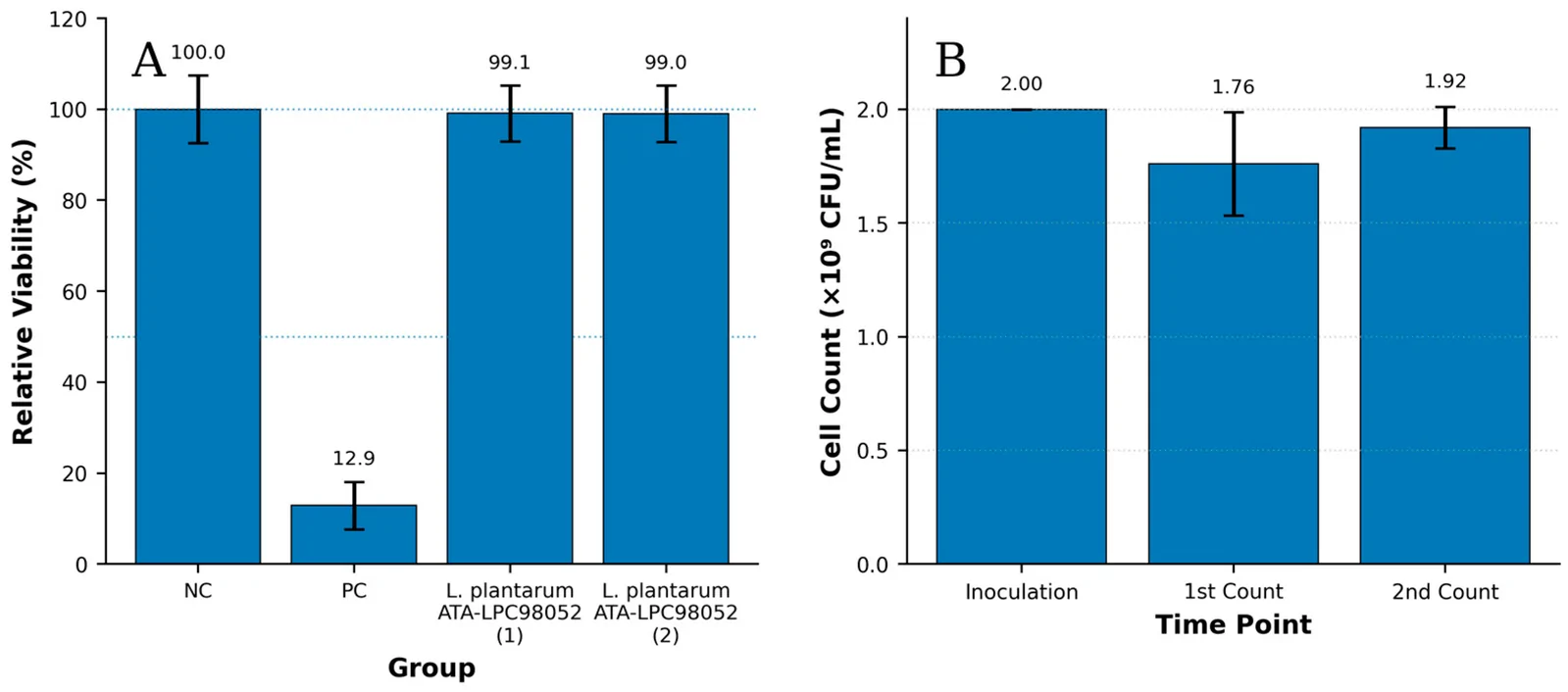
Cytotoxicity and adhesion properties of *Lactiplantibacillus plantarum* ATA-LPC98052. (**A**) Relative viability of Caco-2 cells following exposure to the test strain compared with the negative (NC) and positive (PC) controls. (**B**) Adhesion efficiency of ATA-LPC98052 to Caco-2 cells.

Cytotoxicity analysis using Caco-2 cells demonstrates that ATA-LPC98052 did not adversely affect cell viability. Relative cell viability remained at 99.0% and 98.9% compared with the negative control, which suggests the absence of detectable cytotoxic effects (Figure 2A).

The strain also exhibited robust adhesion to Caco-2 cells, with an adhesion efficiency exceeding 90% following incubation (Figure 2B).

**Figure 3:**
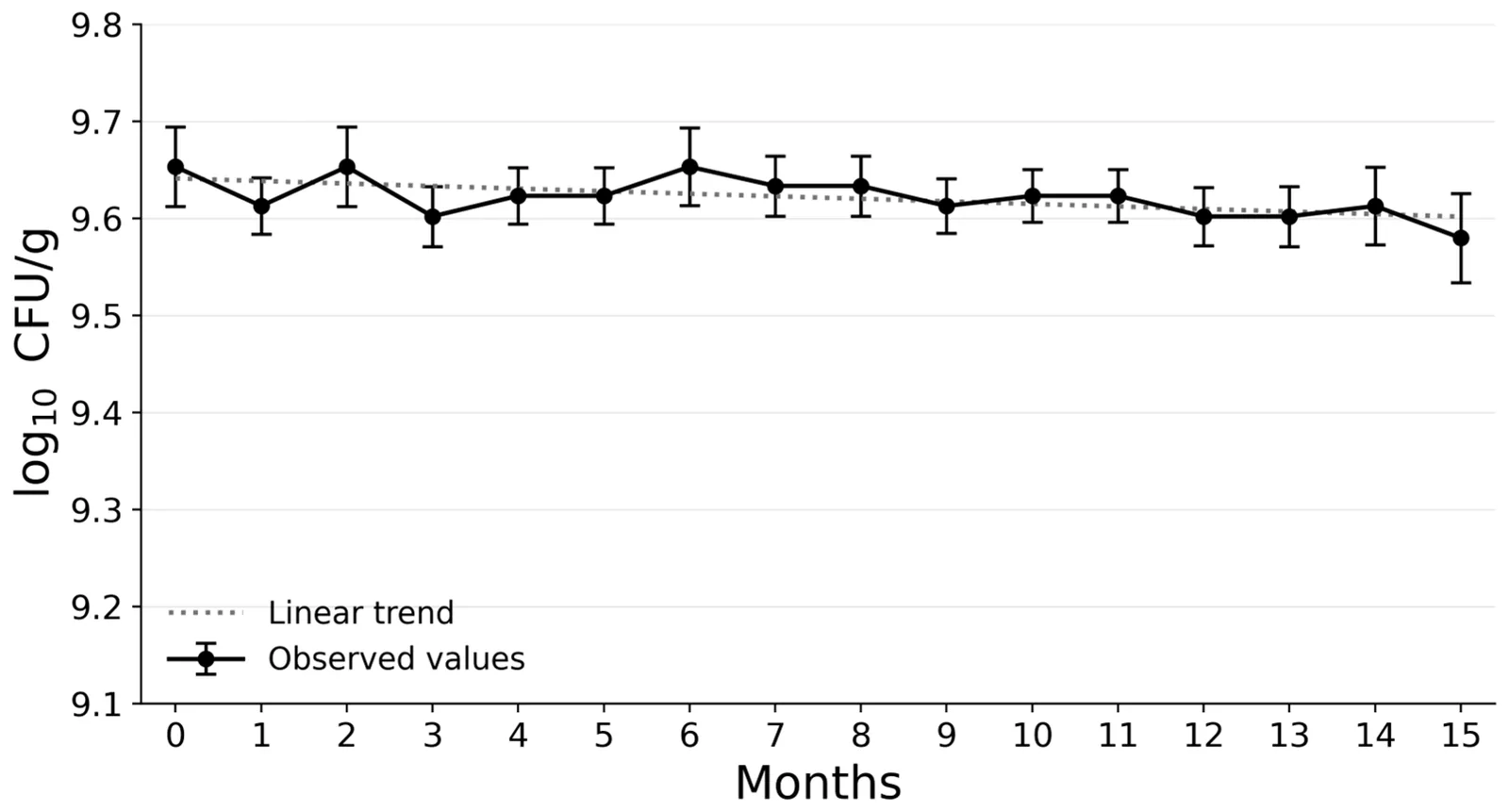
Long-term stability of the lyophilized *Lactiplantibacillus plantarum* ATA-LPC98052 preparation during 15 months of storage at room temperature. culturable cell counts are expressed as log_10_ CFU/g.

Long-term stability testing suggests that the lyophilized preparation maintained culturable counts of approximately 9.6 log_10_ CFU/g throughout the 15-month storage period at room temperature, as no substantial decline in bacterial viability was observed (Figure 3).

### 3.2. In Vivo Subacute Toxicity Clinical Observations

#### 3.2.1. Clinical Observations, Body Weight, and Food Consumption

No mortality or treatment-related clinical signs occurred throughout the 28-day treatment period or the subsequent 14-day recovery period. Daily clinical observations revealed no abnormalities in general appearance, behavior, locomotor activity, or other signs indicative of systemic toxicity following oral administration of *Lactiplantibacillus plantarum* ATA-LPC98052.

**Figure 4:**
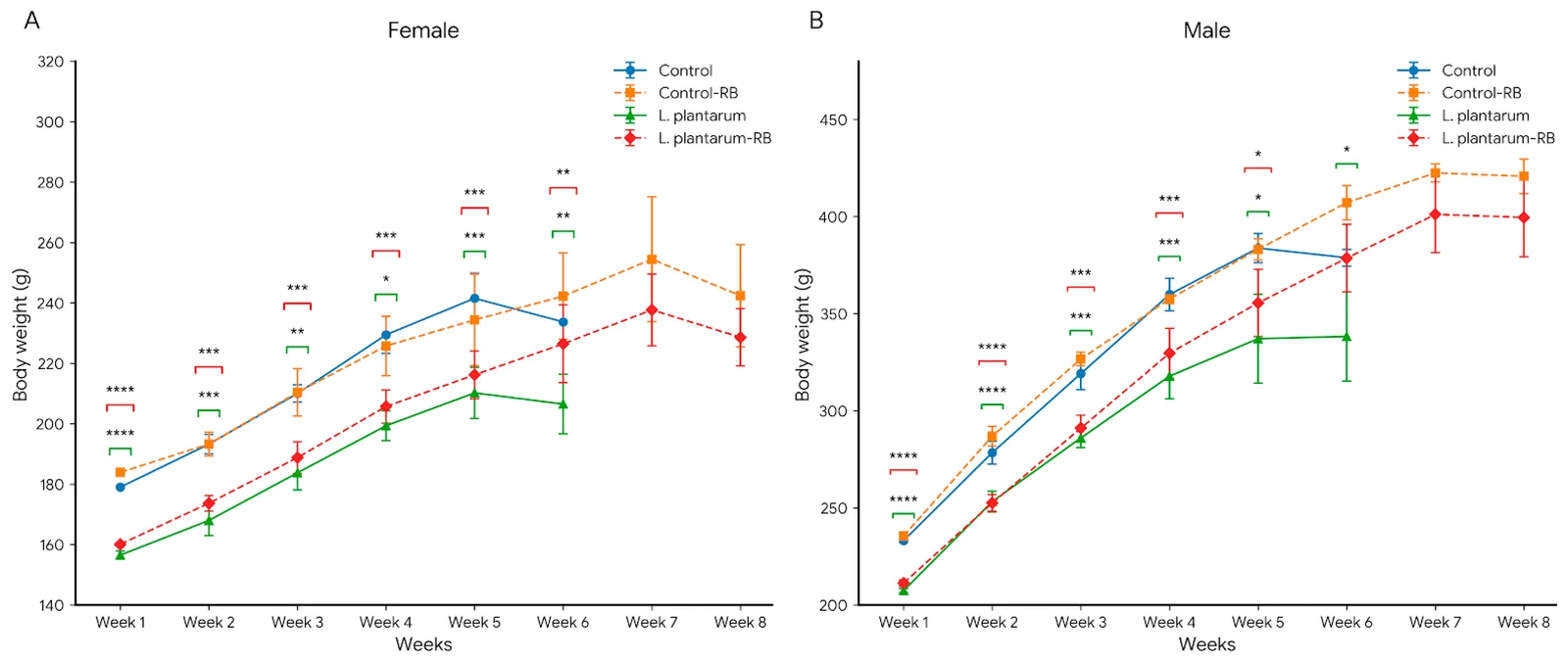
Weekly body weight changes in rats administered *Lactiplantibacillus plantarum* ATA-LPC98052. (**A**) Female rats. (**B**) Male rats. Body weight values are presented as mean ± SD. Control and *L. plantarum* groups were monitored during the treatment period, whereas Control-RB and *L. plantarum*-RB groups were monitored during the recovery period. Statistical comparisons were performed between the corresponding control and treatment groups at each time point. Brackets indicate statistically significant differences between Control vs. *L. plantarum* (Main phase, weeks 1–6) and Control-RB vs. *L. plantarum*-RB (Rebound phase, weeks 1–8): * *p* < 0.05, ** *p* < 0.01, *** *p* < 0.001, **** *p* < 0.0001.

Body weight changes are presented in Figure 4. Both female and male rats demonstrated a progressive increase in body weight throughout the treatment and recovery periods. Although animals receiving *L. plantarum* ATA-LPC98052 exhibited significantly lower absolute body weights than their respective control groups at several time points, body weight increased continuously in all groups during the study, and no treatment-related body weight loss was observed (Figure 4).

Food consumption data are summarized in Table 1. No statistically significant differences were observed between the control and ATA-LPC98052-treated female rats at Day 0 or Day 28. In males, food consumption was significantly lower in the group subsequently assigned to ATA-LPC98052 at baseline (Day 0; *p* = 0.010), whereas no significant difference was observed at Day 28 (*p* = 0.094). Because the baseline difference preceded treatment, it was not considered treatment related.

Overall, oral administration of *Lactiplantibacillus plantarum* ATA-LPC98052 appears not to have been associated with mortality, clinical abnormalities, or treatment-related body weight loss during the study period.

#### 3.2.2. Behavioral and Functional Assessments

Functional assessments indicated the absence of abnormalities within the cutaneous, ocular, and mucosal structures, as well as the respiratory, circulatory, and nervous systems. Furthermore, the absence of clinical indicators—such as tremor, convulsions, diarrhea, lethargy, or coma—may suggest a lack of systemic toxicity.

#### 3.2.3. Hematological Parameters

Hematological findings are summarized in Table 2. Upon the completion of 28 days of oral administration, the female cohort receiving *Lactiplantibacillus plantarum* ATA-LPC98052 exhibited significantly higher total leukocyte (WBC), monocyte (MON), and granulocyte (GRA) counts in comparison to the negative control group, whereas platelet (PLT) counts were significantly lower. No significant differences were observed in lymphocyte count, erythrocyte count, hematocrit, or hemoglobin concentration between the groups.

Throughout the recovery period, the female subjects within the *L. plantarum* group manifested significantly lower WBC, lymphocyte, and granulocyte counts than the recovery control group. In contrast, lymphocyte percentage, monocyte percentage, hemoglobin concentration, and platelet counts were significantly higher in treated animals. No significant differences were detected in erythrocyte count or hematocrit.

In the male cohort, no statistically significant differences were observed between the control and *L. plantarum* groups regarding any hematological parameter at either Day 28 or Day 42.

Overall, the observed hematological alterations were limited to the female cohort. Statistically significant increases in WBC and GRA were observed at the end of the treatment period, followed by statistically significant decreases at the end of the recovery period. In contrast, a statistically significant decrease in PLT was observed at the end of the treatment period, followed by a statistically significant increase at the end of the recovery period. These changes were not accompanied by corresponding clinical or pathological findings.

#### 3.2.4. Biochemical Parameters

Serum biochemical and clotting parameters are summarized in Table 3. Following 28 days of oral administration, female rats receiving *Lactiplantibacillus plantarum* ATA-LPC98052 appeared to manifest a modest but statistically significant increase in serum glucose levels compared with the negative control group; furthermore, no significant differences were observed in liver enzyme activities (ALT, AST, and ALP), renal function markers (urea and creatinine), electrolyte concentrations, lipid profile, total protein, albumin, or clotting time at the end of the treatment period.

During the recovery period, female rats exhibited significantly higher AST activity and a shorter clotting time than the corresponding recovery controls, whereas the remaining biochemical parameters did not differ significantly between groups. CRP concentrations remained below the detection limit in all female groups.

In male rats, no statistically significant differences in serum biochemical parameters were detected following the 28-day treatment period. During recovery, serum potassium, calcium, and total protein concentrations were significantly higher in the *L. plantarum* group than in the recovery controls, whereas all other biochemical parameters, including liver and kidney function markers, remained comparable between groups. CRP concentrations were below the detection limit in all male groups.

Overall, only limited and isolated changes in selected serum biochemical parameters were observed, with no consistent treatment-related pattern across sexes or study periods. Importantly, these alterations were not accompanied by corresponding clinical signs, changes in body weight gain, organ weight alterations, or histopathological lesions, suggesting that they represent incidental physiological variation rather than biologically adverse or treatment-related toxic effects.

#### 3.2.5. Organ Weights and Macroscopic Findings

The summary of relative organ weights is provided in Table 4, in which the assessment encompasses the brain, liver, kidneys, spleen, heart, thymus, adrenal glands, and, where applicable, sex-specific reproductive organs. No statistically significant disparities in the relative weights of any examined organ were observed between the negative control and *Lactiplantibacillus plantarum* ATA-LPC98052-treated cohorts following the 28-day treatment period; similarly, the evaluation of organ weights during the Day 42 recovery period suggests an absence of significant intergroup variation.

Macroscopic necropsy findings are summarized in Table 5. Gross pathological examination revealed no apparent treatment-related lesions in either sex. The isolated findings observed in individual animals appeared to occur in both the control and *L. plantarum* ATA-LPC98052-treated groups, absent any consistent organ-specific or treatment-related pattern.

#### 3.2.6. Histopathological Findings

Histopathological examination of the liver, kidneys, spleen, heart, lungs, gastrointestinal tract, brain, adrenal glands, thymus, and reproductive organs revealed no treatment-related microscopic lesions in either sex following 28 days of oral administration or after the recovery period. Occasional spontaneous background findings were observed in individual animals and were considered incidental, as they occurred with similar frequency in both the control and treated groups.

### 3.3. Gut Microbiota Profiling Following Lactiplantibacillus plantarum ATA-LPC98052 Administration

Fecal microbiota profiles were analyzed longitudinally using the Day 0 and Day 15 samples from the control and probiotic groups. Following quality filtering, DADA2-based denoising, chimera removal, and bacterial taxonomic assignment, the resulting ASV data were used for diversity and differential-abundance analyses.

**Figure 5:**
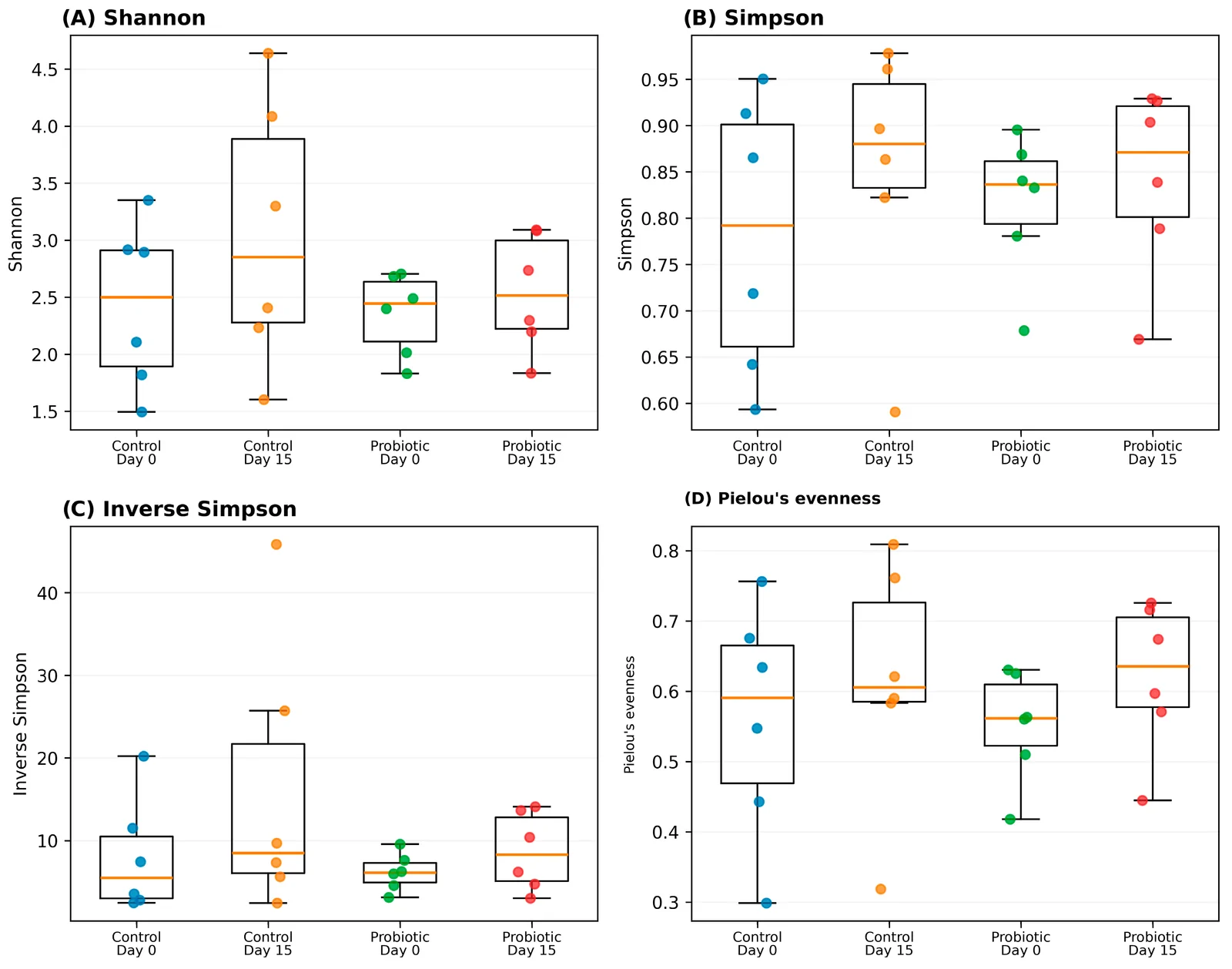
Alpha-diversity indices in control and probiotic groups at Day 0 and Day 15. (**A**) Shannon index, (**B**) Simpson index, (**C**) inverse Simpson index, and (**D**) Pielou’s evenness. Box plots show the median and interquartile range, whiskers indicate the observed distribution, and points represent individual samples. Longitudinal treatment effects were evaluated using linear mixed-effects models with treatment, time, and treatment × time as fixed effects and individual rat identity as a random effect.

Alpha-diversity profiles are shown in Figure 5. Although numerical changes were observed over time within both groups, the treatment × time interaction was not statistically significant for Shannon diversity (beta = −0.427, 95% CI: −1.170 to 0.316, *p* = 0.260), Simpson diversity (beta = −0.045, 95% CI: −0.189 to 0.099, *p* = 0.541), inverse Simpson diversity (beta = −5.615, 95% CI: −13.725 to 2.496, *p* = 0.175), or Pielou’s evenness (beta = 0.015, 95% CI: −0.123 to 0.154, *p* = 0.828). Thus, the change in within-sample diversity from Day 0 to Day 15 did not differ significantly between the probiotic and control groups.

**Figure 6:**
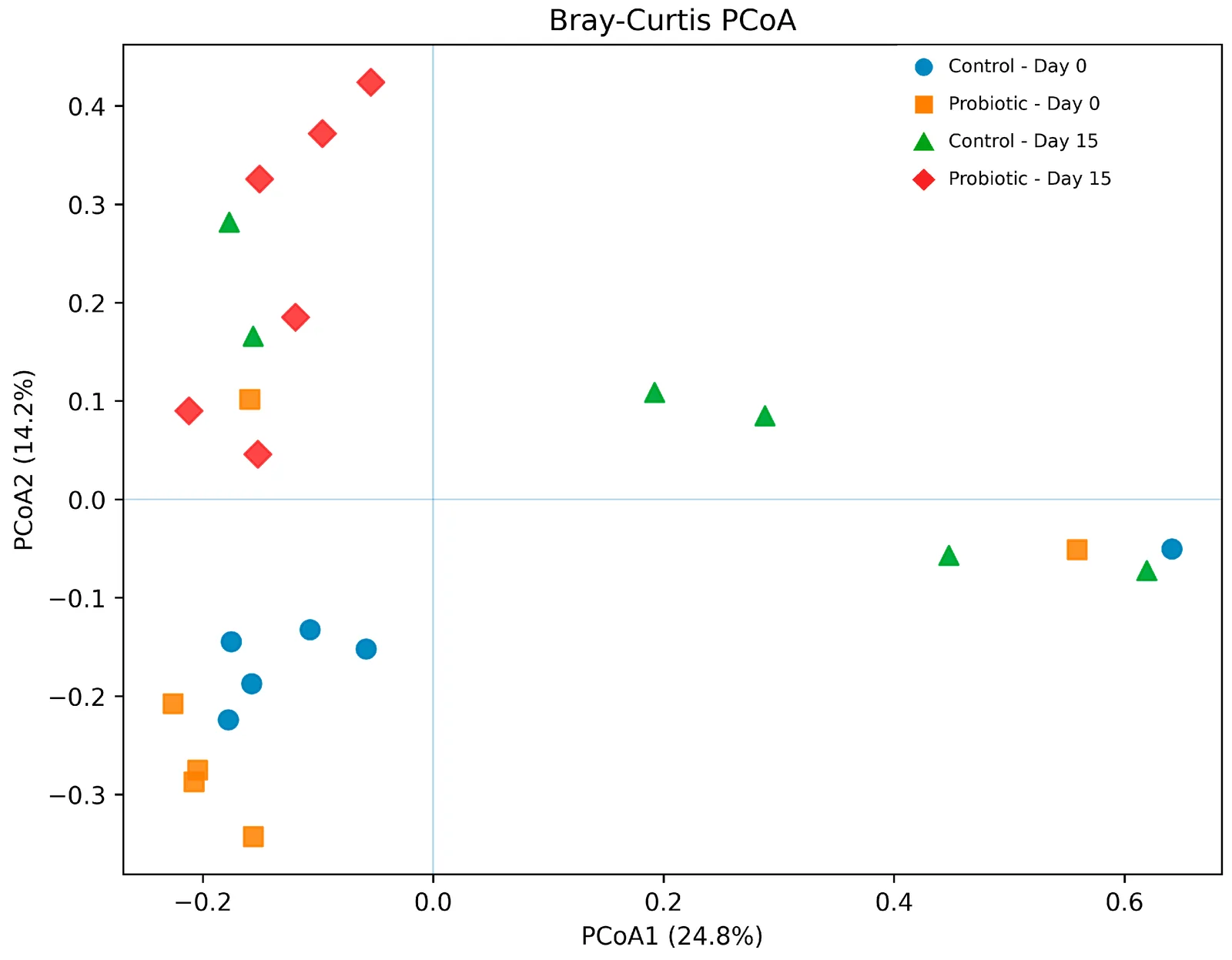
Principal coordinates analysis (PCoA) of Bray–Curtis dissimilarity among control and probiotic samples collected at Day 0 and Day 15. Each point represents one fecal sample. PCoA1 and PCoA2 explain 24.8% and 14.2% of total variation, respectively. Overall group differences were evaluated using PERMANOVA, and dispersion homogeneity was assessed using PERMDISP.

Beta diversity was evaluated using Bray–Curtis dissimilarity and visualized by PCoA (Figure 6). The first two principal coordinates explained 24.8% and 14.2% of the total variation, respectively. When the four group-time combinations were evaluated together (Table 6), overall microbial community composition differed significantly among groups (PERMANOVA: pseudo-F = 1.621, R2 = 0.196, *p* = 0.0226). However, the longitudinal treatment × time interaction was not significant (pseudo-F = 1.072, R2 = 0.043, *p* = 0.5079). PERMDISP did not indicate significant heterogeneity in within-group dispersion (pseudo-F = 0.576, *p* = 0.7287), supporting interpretation of the overall PERMANOVA result as not being driven by a detectable difference in group dispersion.

The overall PERMANOVA used 9999 permutations. For the longitudinal treatment × time analysis, permutations were restricted within individual rat identity.

Formal longitudinal differential-abundance analysis was performed using MaAsLin2 at the genus level. A total of 593 genera were evaluated. No genus remained statistically significant after false-discovery-rate correction at q < 0.05. At the exploratory q < 0.25 threshold, 41 genera showed treatment × time signals. Among the strongest positive interaction coefficients were *Faecalibaculum* (β = 5.318), *Murimonas* (β = 4.341), and *Sellimonas* (β = 4.177), whereas *Prevotella* (β = −7.195), *Alistipes* (β = −5.064), *Dialister* (β = −4.587), *Massiliprevotella* (β = −4.099), and *Neisseria* (β = −4.074) showed negative coefficients. Because none of these associations met the predefined FDR significance threshold of q < 0.05, they were considered exploratory and were not interpreted as confirmed treatment-specific differential abundance. The treatment × time interactions for *Lactobacillus* (β = 1.680, *p* = 0.408, q = 0.719), *Bifidobacterium* (β = −4.616, *p* = 0.146, q = 0.390), *Romboutsia* (β = −0.306, *p* = 0.908, q = 0.994), and *Citrobacter* (β = −3.680, *p* = 0.365, q = 0.681) were also not statistically significant after FDR correction. An exploratory species-level MaAsLin2 analysis is provided in Supplementary Figure S2 and Table S1.

### 3.4. Whole-Genome Sequencing and Genomic Safety Assessment

Whole-genome sequencing confirmed the taxonomic identity of ATA-LPC98052 as *Lactiplantibacillus plantarum*, with no evidence of contamination in the analyzed sample. De novo assembly generated 31 contigs longer than 1000 bp, with a longest contig of 304,743 bp. The genome sequence was deposited in NCBI under BioSample accession SAMN40643420 and BioProject accession PRJNA1092961.

ResFinder screening was performed using minimum thresholds of 80% sequence identity and 60% aligned gene length/coverage and did not identify acquired antimicrobial resistance determinants meeting these criteria. As an additional sensitivity analysis, both the sequence identity and aligned gene length/coverage thresholds were progressively reduced in parallel to approximately 50%; no additional acquired antimicrobial resistance determinants were identified under these lower-stringency conditions. CARD/RGI screening identified two Strict-category homologous matches corresponding to qacJ and vanY of the vanB cluster (Table 7). However, the sequence identities of these matches were low (40.2% for qacJ and 31.93% for vanY), despite high alignment coverage (95.33% and 95.90%, respectively). These matches were therefore interpreted cautiously as low-identity homologous signals rather than direct evidence of phenotypically expressed antimicrobial resistance. Phenotypic antimicrobial susceptibility testing was not performed; therefore, these genomic findings should not be interpreted as a complete antimicrobial susceptibility assessment. Functional gene and pathway annotation did not reveal pathways associated with toxic compound production.

VFDB screening with ABRicate showed a clear stringency-dependent pattern. Nine homologous matches were detected at the lowest threshold (50% identity/50% coverage), whereas only *bsh*, *clpP*, and *hasC* remained at 70% identity, and no matches were detected at identity thresholds of 80% or higher. No hit annotated as Listeria adhesion protein (Lap) was identified under any tested threshold combination. These findings were therefore interpreted cautiously as database homologies rather than direct evidence of pathogenic virulence. Functional gene and pathway annotation did not reveal a complete toxic-compound-associated pathway in the analyzed genome.

## 4. Discussion

The present study offers a comprehensive preclinical safety assessment of *Lactiplantibacillus plantarum* ATA-LPC98052 through in vitro characterization, whole-genome sequencing, a 28-day repeated-dose oral toxicity study, and gut microbiota analysis. Overall, ATA-LPC98052 administration appears not to be associated with mortality, overt clinical toxicity, consistent target organ toxicity, significant relative organ weight changes, or widespread macroscopic pathological findings. Although statistically significant differences were detected in some hematological and biochemical parameters, these changes appear to be mostly sex-, time-, or study phase-specific and may not constitute a consistent toxicity pattern supported by organ weight and necropsy findings. Although *L. plantarum* has an established history of use, QPS/GRAS status does not guarantee the safety of every strain. Therefore, the repeated-dose study was conducted as a supplementary precautionary assessment of the soil-derived ATA-LPC98052 strain, rather than as a routine FAO/WHO requirement or a formal OECD-compliant study.

For a microorganism to be considered probiotic, it must be identified at the strain level, exhibit resistance to gastrointestinal transit conditions, possess the potential to interact with the intestinal epithelium, and meet fundamental safety criteria [21]. In this study, ATA-LPC98052 appears to produce no hemolysis zone in ten parallel replicates, leading to its classification as γ-hemolytic. This finding is consistent with the non-hemolytic profiles reported for *L. plantarum* strains Q180, ZJ316, and N13 [22,23,24]. Therefore, the result obtained can be considered an important in vitro safety indicator supporting the absence of hemolytic potential in ATA-LPC98052; however, it may not be interpreted as definitive evidence that excludes all pathogenicity risks on its own. Moreover, whole-genome sequencing (WGS) appears to confirm the identity of ATA-LPC98052 and suggests the absence of acquired antimicrobial resistance genes, thereby complementing the phenotypic safety assessment. Nevertheless, standardized phenotypic MIC testing remains warranted to further support the safety profile in accordance with current EFSA-FEEDAP recommendations.

The strain exhibited 68% viability at pH 1.5 and 88% viability at pH 5.0; it further maintained ≥90% viability across the 0.1–0.5% bile salt range, which suggests a significant resistance to the acidic environment and bile stress conditions encountered during gastrointestinal transit. Similar acid and bile tolerance profiles have been reported for different *L. plantarum* strains such as BG24 and JS21 [5]. This adaptive capacity may be attributed to the plastic structure of the *L. plantarum* genome, which facilitates the adaptation to diverse environmental niches [1]. The high adhesion rate observed in Caco-2 cells together with approximately 99% cell viability may suggest that the strain possesses the potential to interact with intestinal epithelial surfaces and does not appear to be cytotoxic under the tested conditions [14,25]. In support of this finding, other studies in the literature also show that postbiotic formulations restore the viability of healthy colon fibroblast cells exposed to toxicity and repair cellular damage [26]. These strain-specific properties may be relevant to intestinal health. Acid and bile tolerance may support gastrointestinal survival, whereas Caco-2 adhesion suggests potential epithelial interaction. Descriptive changes in Lactobacillus, Bifidobacterium, Romboutsia, and Citrobacter abundance suggested possible microbiota modulation; however, these taxa did not show FDR-significant treatment × time associations and should therefore be interpreted as exploratory. Nevertheless, in vitro adhesion data are not direct evidence of in vivo colonization and must be confirmed in future animal or human studies [27]. Furthermore, the maintenance of 9.6 log_10_ CFU/g throughout the 15-month room temperature stability study is consistent with the stability reported for *L. plantarum* 299v formulations [28], supporting the strong technological stability profile of the ATA-LPC98052 preparation [29,30]. The sodium alginate–inulin protective matrix may have contributed to the preservation of culturable counts during storage. However, because chain formation and dechaining were not evaluated microscopically, their possible influence on CFU enumeration cannot be excluded. Although plate counting remains the standard method for assessing probiotic viability, it does not detect viable but non-culturable (VBNC) cells. Future studies could therefore complement CFU enumeration with flow cytometry-based viability assays to provide a more comprehensive evaluation of long-term stability.

In the 28-day repeated-dose study adapted from selected principles of OECD Test Guideline 407, administration of ATA-LPC98052 at 1.2 × 10^11^ CFU/kg/day did not yield the occurrence of mortality or overt clinical adverse manifestations. This result is consistent with the general safety profiles reported in repeated-dose safety studies conducted with *L. plantarum* strains such as HD1 [31] and OLL2712 [32]. In the present study, lower absolute body weights were observed in the *L. plantarum*-administered groups compared with the control groups at certain weeks. However, all groups continued to gain weight throughout the study period, with no treatment-related weight loss, mortality, or clinical deterioration. Therefore, body weight differences may not be solely indicative of systemic toxicity. The absolute weight differences present from the early phase suggest that the evaluation of baseline inter-group variability is also warranted. Similarly, although statistically significant differences in feed intake were detected at certain time points, these changes did not display a consistent pattern across sex, period, or time and therefore do not unequivocally substantiate a clear toxicological response.

Statistically significant hematological variations were documented predominantly in female rats; specifically, elevated white blood cell (WBC), monocyte, and granulocyte counts, alongside diminished platelet counts, were observed on day 28. The divergent trajectory of alterations in certain leukocyte-related parameters during the recovery period, in conjunction with the absence of statistically significant hematological modifications in male rats, suggests that these findings may not substantiate persistent or progressive hematotoxicity. Nevertheless, because the study was not specifically designed or powered to detect sex-dependent effects, the female-specific findings should be interpreted with caution. Moreover, the magnitude of these changes was generally comparable with published historical reference data for Wistar rats used in repeated-dose toxicity studies, supporting the interpretation that they most likely reflect biological variability rather than treatment-related hematotoxicity, although historical reference data should be interpreted as supportive rather than replacing concurrent controls. The absence of corroborating evidence from relative organ weights or macroscopic findings appears to preclude the interpretation of these isolated changes as indicators of overt toxicity. Furthermore, the affected biochemical parameters were generally comparable with published physiological reference ranges for Wistar rats and showed no consistent pattern across sexes or study phases, supporting the conclusion that these isolated findings were not toxicologically adverse [33]. Similarly, in OLL2712 [32] and N13 [23], studies, certain parameter changes were evaluated as non-dose-related, low-magnitude, or non-adverse. Nevertheless, the platelet changes observed in females should be confirmed in future studies using individual animal data and, if necessary, longer follow-up.

When biochemical and coagulation parameters were evaluated, no consistent hepatic, renal, metabolic, or coagulopathic toxicity pattern was observed. Although increases in glucose on day 28 and in AST during the recovery period were detected in females, no parallel deterioration was observed in ALT, ALP, bilirubin, urea, creatinine, or CRP parameters. In males, transient elevations in potassium, calcium, and total protein were observed solely during the recovery period; however, such fluctuations may lack clinical significance. The absence of corroborating evidence from relative organ weights or macroscopic evaluations suggests that these isolated deviations may not signify overt target organ toxicity. Although the metabolic and immunoregulatory potential of *L. plantarum* strains has been reported [8,9,10], the present study was designed primarily as a safety assessment. Accordingly, complementary mechanistic endpoints, such as inflammatory cytokine profiling, microbial metabolite analyses (e.g., short-chain fatty acids), and host immune or metabolic biomarkers, were not evaluated. As these approaches are increasingly incorporated into contemporary probiotic research to connect microbial changes with host function, the present findings should be interpreted as evidence of safety and microbiota modulation rather than confirmation of specific biological mechanisms.

In terms of relative organ weights, no significant differences were detected in either female or male rats, supporting the absence of overt organ-level toxicity. Furthermore, no statistically significant alterations were identified within the male reproductive apparatus. Macroscopic necropsy findings in the kidney and spleen were limited and sporadic, recorded in a small number of animals. The fact that these findings were observed both in control and treatment groups and did not display a consistent sex, period, or treatment distribution suggests that they do not constitute a treatment-related pathological pattern.

The revised longitudinal 16S rRNA analysis provided a more conservative interpretation of microbiota changes following ATA-LPC98052 administration. Previous studies have demonstrated that *L. plantarum* strains may modulate gut microbial communities in a strain-dependent manner [7,10]. In the present study, however, treatment × time interactions were not statistically significant for the Shannon, Simpson, inverse Simpson, or Pielou’s evenness indices, indicating that temporal changes in alpha diversity did not differ significantly between the probiotic and control groups. Similarly, although Bray–Curtis-based PERMANOVA detected an overall difference among the four group–time combinations, the longitudinal treatment × time interaction was not statistically significant, and PERMDISP indicated no significant heterogeneity in within-group dispersion. Formal longitudinal differential-abundance analysis using MaAsLin2 likewise did not identify taxa that remained statistically significant after false-discovery-rate correction at q < 0.05. Accordingly, the taxonomic changes observed in relative-abundance profiles should be regarded as descriptive or exploratory rather than as confirmed treatment-specific effects. These findings highlight the importance of longitudinal modeling and multiple-testing correction when interpreting microbiome compositional data [34,35]. Furthermore, because the present analysis was based on short-term V3–V4 16S rRNA profiling, it does not provide sufficient resolution to establish strain-level colonization or to directly link compositional variation to functional metabolic or immunological outcomes. Future studies integrating shotgun metagenomics, metabolomics, cytokine profiling, and strain-specific colonization approaches will be required to determine whether microbiota changes associated with ATA-LPC98052 translate into measurable host effects.

The expanded whole-genome sequencing analysis provided additional information for the strain-level safety assessment of ATA-LPC98052. Genome analysis confirmed the identity of the isolate as *Lactiplantibacillus plantarum* with no evidence of contamination. ResFinder screening did not identify acquired antimicrobial resistance determinants at the predefined thresholds of ≥80% sequence identity and ≥60% gene coverage. CARD/RGI analysis nevertheless identified Strict-category homologous matches corresponding to *qacJ* and *vanY* of the *vanB* cluster. However, these matches exhibited low sequence identities to the respective reference determinants (40.2% and 31.93%, respectively), despite high alignment coverage, and were therefore interpreted cautiously as low-identity homologous signals rather than direct evidence of functional antimicrobial resistance. This interpretation is consistent with previous observations that database screening at different stringency levels may identify partial or low-confidence AMR-associated homologs that require contextual evaluation [36]. VFDB screening showed a clear stringency-dependent pattern, with several homologous matches detected under lower-stringency conditions but progressively fewer matches retained as identity and coverage thresholds increased. No VFDB matches were detected at identity thresholds of 80% or higher, and no hit annotated as Listeria adhesion protein (Lap) was identified under any tested threshold combination. Because sequence homology to proteins catalogued in virulence databases does not by itself establish pathogenic potential, these findings were interpreted cautiously in the context of sequence similarity, screening stringency, and the overall phenotypic safety profile of the strain [37]. Together with the γ-hemolytic phenotype, absence of detectable cytotoxicity, and lack of treatment-related adverse findings in the subacute toxicity study, the genomic findings support a favorable preliminary safety profile for ATA-LPC98052. Nevertheless, standardized phenotypic MIC testing was not performed. Therefore, genomic screening should not be considered a substitute for phenotypic antimicrobial susceptibility testing, and the antimicrobial susceptibility profile of ATA-LPC98052 remains to be confirmed experimentally as part of subsequent strain-validation studies [30].

### Limitations

Although DADA2-based ASV inference and the SILVA 138 reference database improve taxonomic resolution, genus-level assignments based on the V3-V4 region of the 16S rRNA gene should be interpreted with caution. The microbiota analysis was short-term, and no FDR-significant genus-level treatment × time associations were identified. Accordingly, the observed exploratory signals cannot be directly linked to functional host responses or to strain-specific colonization. Future studies incorporating genus-focused differential-abundance analysis, shotgun metagenomics, strain-specific quantitative assays, metabolomics, and immunological measurements would allow more definitive interpretation of treatment-associated microbiota effects.

Similarly, genomic detection of resistance- or virulence-associated homologs does not establish functional phenotype. Standardized phenotypic MIC testing, assessment of genomic context and mobile genetic elements, and targeted functional validation of relevant high-confidence homologs would strengthen the safety interpretation. Metabolic safety assays (e.g., D-lactate and biogenic amine production) and regulatory genotoxicity testing were also beyond the scope of the present study.

## 5. Conclusions

ATA-LPC98052 demonstrated favorable probiotic-related characteristics and an overall supportive preclinical safety profile under the tested conditions. The revised longitudinal 16S rRNA analysis did not demonstrate statistically significant treatment-specific changes in alpha diversity or overall Bray–Curtis community structure, and genus-level MaAsLin2 analysis did not identify taxa remaining significant after FDR correction at q < 0.05; therefore, the microbiota findings should be regarded as exploratory. Genomic screening did not identify acquired antimicrobial resistance determinants meeting the predefined ResFinder thresholds, although low-identity CARD/RGI homologs and stringency-dependent VFDB homologous matches were detected and require cautious contextual interpretation. Further studies incorporating phenotypic MIC testing, functional genomic validation, and higher-resolution microbiome approaches are warranted to strengthen the overall safety and functional characterization of ATA-LPC98052.

## Figures and Tables

**Table 1:** Food consumption in rats administered *Lactiplantibacillus plantarum* ATA-LPC98052.

Parameters	Females			Males		
	NC	*L. plantarum*	*p*	NC	*L. plantarum*	*p*
Food consumption (g/animal), Day 0	16.5 ± 2.1	15.1 ± 1.5	0.263	23.7 ± 0.9	21.8 ± 0.9	**0.010**
Food consumption (g/animal), Day 28	18.7 ± 2.6	17.4 ± 0.9	0.340	24.2 ± 1.6	21.7 ± 2.4	0.094

Results are expressed as the arithmetic mean ± standard deviation; statistical evaluation of the variations between the negative control (NC) and *L. plantarum* ATA-LPC98052-treated cohorts was conducted independently for each sex. Bold *p* values indicate *p* < 0.05.

**Table 2:** Hematological parameters in female and male rats following repeated oral administration of *Lactiplantibacillus plantarum* ATA-LPC98052.

Parameter (Unit)	Main NC Day 28	Main *L. plantarum* Day 28	*p*	Recovery NC Day 42	Recovery *L. plantarum* Day 42	*p*
**Female**						
WBC (Leukocytes; 10^9^/L)	12.3 ± 0.7	19.6 ± 2.4	**0.006**	25.9 ± 4.6	17.5 ± 4.7	**0.021**
LYM (Lymphocytes; 10^9^/L)	8.8 ± 1.5	13.9 ± 4.6	0.067	15.5 ± 2.8	11.3 ± 2.4	**0.035**
LYM (Lymphocytes; %)	65.9 ± 6.2	62.5 ± 0.6	0.288	59.6 ± 3.6	66.4 ± 1.1	**0.011**
MON (Monocytes; 10^9^/L)	0.6 ± 0.1	0.9 ± 0.1	**0.003**	1.2 ± 0.3	1.0 ± 0.2	0.255
MON (Monocytes; %)	5.3 ± 0.6	4.9 ± 0.4	0.255	4.7 ± 0.2	6.0 ± 0.9	**0.030**
GRA (Granulocytes; 10^9^/L)	3.8 ± 1.2	7.7 ± 2.4	**0.018**	9.1 ± 1.9	5.2 ± 2.3	**0.020**
GRA (Granulocytes; %)	28.8 ± 6.3	32.5 ± 0.9	0.261	35.3 ± 3.6	27.2 ± 1.0	**0.006**
HGB (Hemoglobin; g/dL)	14.3 ± 0.5	14.5 ± 0.7	0.619	14.6 ± 0.3	15.0 ± 0.1	**0.038**
RBC (Erythrocytes; 10^12^/L)	8.29 ± 0.64	8.66 ± 0.61	0.377	8.27 ± 0.24	8.58 ± 0.26	0.086
HCT (Hematocrit; %)	39.4 ± 1.1	40.4 ± 1.3	0.227	39.1 ± 0.9	39.8 ± 0.3	0.161
PLT (Platelets; 10^9^/L)	959.8 ± 107.2	357.8 ± 275.8	**0.005**	212.8 ± 87.0	758.0 ± 329.0	**0.019**
**Male**						
WBC (Leukocytes; 10^9^/L)	33.3 ± 4.2	35.6 ± 9.4	0.637	29.2 ± 9.9	29.7 ± 4.5	0.922
LYM (Lymphocytes; 10^9^/L)	20.0 ± 2.3	22.0 ± 5.4	0.478	17.4 ± 5.8	18.0 ± 2.1	0.836
LYM (Lymphocytes; %)	59.9 ± 2.6	61.9 ± 3.5	0.337	59.5 ± 0.8	60.5 ± 2.6	0.450
MON (Monocytes; 10^9^/L)	1.7 ± 0.2	1.6 ± 0.1	0.366	1.5 ± 0.4	1.5 ± 0.2	1.000
MON (Monocytes; %)	5.4 ± 0.2	5.4 ± 0.5	1.000	5.3 ± 0.1	5.2 ± 0.1	0.182
GRA (Granulocytes; 10^9^/L)	11.6 ± 2.1	11.8 ± 3.7	0.920	10.3 ± 3.6	10.3 ± 2.3	1.000
GRA (Granulocytes; %)	34.7 ± 2.8	32.7 ± 4.0	0.390	35.1 ± 1.1	34.3 ± 2.7	0.565
HGB (Hemoglobin; g/dL)	15.1 ± 0.6	15.4 ± 0.3	0.357	14.9 ± 0.6	15.2 ± 0.3	0.357
RBC (Erythrocytes; 10^12^/L)	8.57 ± 0.34	8.76 ± 0.27	0.358	8.69 ± 0.53	9.06 ± 0.39	0.247
HCT (Hematocrit; %)	40.8 ± 1.5	42.0 ± 0.6	0.155	39.9 ± 0.7	40.7 ± 1.1	0.214
PLT (Platelets; 10^9^/L)	266.6 ± 135.9	295.6 ± 250.2	0.827	129.3 ± 80.2	162.3 ± 30.5	0.486

Data are presented as the mean ± standard deviation (*n* = 5 per sex per group). NC: negative control; Main, animals evaluated at the end of the 28-day treatment period (Day 28); RB: Recovery, animals evaluated after the 14-day recovery period (Day 42). BDL: below detection limit; NA: not applicable. *p*-values compare NC vs. *L. plantarum* within the same sex and time point. Bold *p* values indicate *p* < 0.05.

**Table 3:** Serum biochemical and clotting parameters in female and male rats following repeated oral administration of *Lactiplantibacillus plantarum* ATA-LPC98052.

Female						
Parameter	Main NC	Main *L. plantarum*	*p*	Recovery NC	Recovery *L. plantarum*	*p*
ALP (U/L)	107.38 ± 22.33	122.62 ± 34.87	0.438	73.16 ± 20.76	91.32 ± 41.20	0.413
ALT (U/L)	56.28 ± 15.89	48.68 ± 7.97	0.377	54.34 ± 7.08	63.93 ± 17.56	0.365
AST (U/L)	129.38 ± 33.63	107.44 ± 12.89	0.230	91.33 ± 5.48	110.03 ± 9.32	**0.019**
Urea (mg/dL)	44.60 ± 11.73	45.80 ± 10.33	0.868	49.75 ± 1.24	49.46 ± 11.63	0.958
Creatinine (mg/dL)	0.65 ± 0.04	0.64 ± 0.03	0.667	0.68 ± 0.04	0.66 ± 0.03	0.399
Total bilirubin (mg/dL)	0.14 ± 0.03	0.43 ± 0.38	0.163	BDL	BDL	NA
CRP (mg/L)	BDL	BDL	NA	BDL	BDL	NA
Sodium (mmol/L)	141.04 ± 2.37	142.29 ± 1.09	0.328	141.92 ± 0.92	138.96 ± 2.52	0.056
Potassium (mmol/L)	4.48 ± 0.35	3.90 ± 0.50	0.070	3.89 ± 0.46	3.76 ± 0.49	0.677
Calcium (mg/dL)	9.55 ± 0.31	9.46 ± 0.19	0.598	9.58 ± 0.25	9.42 ± 0.24	0.332
Cholesterol (mg/dL)	57.45 ± 4.36	63.95 ± 6.34	0.100	69.75 ± 8.32	70.41 ± 7.54	0.899
Glucose (mg/dL)	108.47 ± 5.08	117.66 ± 5.48	**0.049**	150.25 ± 21.93	147.07 ± 29.56	0.852
Triglyceride (mg/dL)	63.03 ± 20.05	50.45 ± 7.67	0.246	56.02 ± 5.85	76.55 ± 36.55	0.280
Total protein (g/dL)	5.77 ± 0.26	6.12 ± 0.28	0.075	6.32 ± 0.42	6.31 ± 0.29	0.966
Albumin (g/dL)	4.41 ± 0.22	4.31 ± 0.23	0.502	4.41 ± 0.10	4.49 ± 0.38	0.672
Clotting time (s)	23.50 ± 2.40	24.00 ± 1.50	0.705	31.40 ± 1.20	29.00 ± 0.80	**0.009**
**Male**						
**Parameter**	**Main NC**	**Main *L. plantarum***	***p***	**Recovery NC**	**Recovery *L. plantarum***	***p***
ALP (U/L)	144.16 ± 32.60	132.84 ± 9.39	0.491	146.24 ± 36.93	142.22 ± 20.61	0.838
ALT (U/L)	50.98 ± 8.79	47.54 ± 7.79	0.531	53.86 ± 5.20	56.84 ± 10.79	0.599
AST (U/L)	104.84 ± 11.31	107.42 ± 37.98	0.890	88.64 ± 11.64	98.50 ± 11.21	0.210
Urea (mg/dL)	38.78 ± 5.87	43.98 ± 7.01	0.240	51.16 ± 8.35	52.30 ± 9.04	0.841
Creatinine (mg/dL)	0.60 ± 0.01	0.62 ± 0.03	0.218	0.63 ± 0.02	0.62 ± 0.04	0.635
Total bilirubin (mg/dL)	0.23 ± 0.18	0.21 ± 0.13	0.846	0.14 ± 0.03	0.14 ± 0.01	1.000
CRP (mg/L)	BDL	BDL	NA	BDL	BDL	NA
Sodium (mmol/L)	141.69 ± 0.87	141.22 ± 0.82	0.405	141.64 ± 2.01	143.08 ± 1.37	0.227
Potassium (mmol/L)	4.26 ± 0.19	4.43 ± 0.40	0.425	4.30 ± 0.06	4.64 ± 0.14	**0.003**
Calcium (mg/dL)	9.84 ± 0.17	9.69 ± 0.32	0.390	9.75 ± 0.17	10.18 ± 0.25	**0.015**
Cholesterol (mg/dL)	59.79 ± 6.66	58.98 ± 12.12	0.900	59.20 ± 5.73	60.49 ± 9.54	0.803
Glucose (mg/dL)	151.69 ± 12.75	148.45 ± 4.66	0.620	158.17 ± 9.47	150.26 ± 13.73	0.324
Triglyceride (mg/dL)	97.91 ± 35.84	85.60 ± 31.99	0.583	81.57 ± 9.29	78.99 ± 17.79	0.783
Total protein (g/dL)	6.30 ± 0.39	6.44 ± 0.45	0.614	6.39 ± 0.23	6.97 ± 0.39	**0.026**
Albumin (g/dL)	4.59 ± 0.51	4.70 ± 0.07	0.657	5.95 ± 0.29	6.03 ± 0.47	0.756
Clotting time (s)	27.90 ± 1.10	28.30 ± 1.40	0.630	26.80 ± 1.90	24.80 ± 0.70	0.123

Data are presented as mean ± SD (*n* = 5 per sex per group). NC: negative control; Main, animals evaluated at the end of the 28-day treatment period (Day 28); RB: Recovery, animals evaluated after the 14-day recovery period (Day 42). BDL: below detection limit; NA: not applicable. *p* values compare NC vs. *L. plantarum* within the same sex and time point. Bold *p* values indicate *p* < 0.05.

**Table 4:** Relative organ weights in female and male rats following repeated oral administration of *Lactiplantibacillus plantarum* ATA-LPC98052.

Organ	Main Groups (Day 28)			Recovery Groups (Day 42)		
Organ	NC	*L. plantarum*	*p*	NC	*L. plantarum*	*p*
**Female**						
Brain	0.0078 ± 0.0006	0.0086 ± 0.0007	0.089	ND	ND	NA
Spleen	0.0028 ± 0.0005	0.0028 ± 0.0002	1.000	0.0028 ± 0.0002	0.0029 ± 0.0005	0.694
Liver	0.0273 ± 0.0021	0.0291 ± 0.0014	0.155	0.0268 ± 0.0008	0.0273 ± 0.0019	0.609
Kidneys	0.0069 ± 0.0006	0.0066 ± 0.0002	0.339	0.0064 ± 0.0002	0.0066 ± 0.0005	0.442
Adrenal glands	0.0003 ± 0.0001	0.0003 ± 0.0001	1.000	0.0003 ± 0.0000	0.0003 ± 0.0000	1.000
Heart	0.0039 ± 0.0005	0.0042 ± 0.0004	0.327	0.0036 ± 0.0003	0.0036 ± 0.0003	1.000
Thymus	0.0037 ± 0.0016	0.0031 ± 0.0003	0.453	0.0022 ± 0.0003	0.0025 ± 0.0002	0.105
**Male**						
Brain	0.0051 ± 0.0002	0.0057 ± 0.0005	0.053	ND	ND	NA
Spleen	0.0024 ± 0.0002	0.0025 ± 0.0004	0.635	0.0021 ± 0.0003	0.0023 ± 0.0006	0.530
Liver	0.0315 ± 0.0021	0.0319 ± 0.0018	0.755	0.0307 ± 0.0007	0.0317 ± 0.0015	0.235
Kidneys	0.0066 ± 0.0004	0.0064 ± 0.0004	0.452	0.0058 ± 0.0002	0.0066 ± 0.0008	0.088
Adrenal glands	0.0002 ± 0.0000	0.0002 ± 0.0000	1.000	0.0001 ± 0.0000	0.0002 ± 0.0000	NA
Heart	0.0036 ± 0.0006	0.0035 ± 0.0002	0.738	0.0029 ± 0.0001	0.0030 ± 0.0001	0.182
Thymus	0.0020 ± 0.0004	0.0020 ± 0.0002	1.000	0.0016 ± 0.0001	0.0018 ± 0.0003	0.218
Testes	0.0090 ± 0.0004	0.0102 ± 0.0013	0.109	ND	ND	NA
Epididymides	0.0029 ± 0.0002	0.0032 ± 0.0005	0.266	ND	ND	NA
Prostate, seminal vesicles and coagulating glands	0.0050 ± 0.0008	0.0049 ± 0.0006	0.829	ND	ND	NA

Data are presented as mean ± SD (*n* = 5 per sex per group). NC, negative control; Main, animals euthanized after the 28-day treatment period; RB, recovery group, animals maintained for a 14-day treatment-free recovery period and euthanized on Day 42; ND, not determined; NA, not applicable. *p* values compare NC and *L. plantarum* groups within the same sex and study phase. Values in bold indicate statistical significance (*p* < 0.05).

**Table 5:** Macroscopic necropsy findings following repeated oral administration of *Lactiplantibacillus plantarum* ATA-LPC98052. (Number of animals with finding/total number of animals in group).

Tissue/Organ	Female				Male			
	NC		*L. plantarum*		NC		*L. plantarum*	
	Main	RB	Main	RB	Main	RB	Main	RB
Brain (cerebrum, cerebellum and pons included)	0/5	X	0/5	X	0/5	X	0/5	X
Spinal cord	0/5	X	0/5	X	0/5	X	0/5	X
Eye	0/5	X	0/5	X	0/5	X	0/5	X
Stomach	0/5	0/5	0/5	0/5	0/5	0/5	0/5	0/5
Small intestine (including Peyer’s patches)	0/5	0/5	0/5	0/5	0/5	0/5	0/5	0/5
Large intestine	0/5	0/5	0/5	0/5	0/5	0/5	0/5	0/5
Liver	0/5	0/5	0/5	0/5	0/5	0/5	0/5	0/5
Kidneys	0/5	0/5	0/5	1/5	0/5	0/5	0/5	2/5
Adrenal glands	0/5	0/5	0/5	0/5	0/5	0/5	0/5	0/5
Spleen	1/5	0/5	0/5	1/5	0/5	2/5	1/5	0/5
Heart	0/5	0/5	0/5	0/5	0/5	0/5	0/5	0/5
Thymus	0/5	0/5	0/5	0/5	0/5	0/5	0/5	0/5
Thyroid (with larynx)	0/5	X	0/5	X	0/5	X	0/5	X
Trachea	0/5	X	0/5	X	0/5	X	0/5	X
Lung	0/5	0/5	0/5	0/5	0/5	0/5	0/5	0/5
Urinary bladder	0/5	X	0/5	X	0/5	X	0/5	X
Mesenteric lymph node	0/5	0/5	0/5	0/5	0/5	0/5	0/5	0/5
Axillary lymph node	0/5	X	0/5	X	0/5	X	0/5	X
Sciatic nerve	0/5	X	0/5	X	0/5	X	0/5	X
Skeletal muscle	0/5	X	0/5	X	0/5	X	0/5	X
Femur	0/5	X	0/5	X	0/5	X	0/5	X
Bone marrow aspirate	LA	X	LA	X	LA	X	LA	X
Ovary	0/5	X	0/5	X	-	-	-	-
Uterus and cervix	0/5	X	0/5	X	-	-	-	-
Vagina	0/5	X	0/5	X	-	-	-	-
Vaginal smear	LA	X	LA	X	-	-	-	-
Testes	-	-	-	-	0/5	X	0/5	X
Epididymides	-	-	-	-	0/5	X	0/5	X
Prostate, seminal vesicles and coagulating glands	-	-	-	-	0/5	X	0/5	X
Lesion (if present)	0/5	0/5	0/5	0/5	0/5	0/5	0/5	0/5

Data are expressed as the ratio of animals exhibiting macroscopic findings to the total number of subjects in the group. Abbreviations: NC, negative control; Main, animals euthanized after the 28-day treatment period; RB, recovery group (euthanized after a 14-day recovery period); X, no macroscopic finding detected; LA, tissue not collected; -, organ not applicable due to sex-specific anatomy.

**Table 6:** Treatment × time interaction effects for alpha-diversity indices in the longitudinal linear mixed-effects models.

Parameter	β	95% CI	*p* Value
Shannon	−0.427	−1.170 to 0.316	0.260
Simpson	−0.045	−0.189 to 0.099	0.541
Inverse Simpson	−5.615	−13.725 to 2.496	0.175
Pielou’s evenness	0.015	−0.123 to 0.154	0.828

Beta, model coefficient for the treatment × time interaction; CI, confidence interval. No interaction term reached *p* < 0.05.

**Table 7:** CARD/RGI Strict-category homologous matches identified in the ATA-LPC98052 genome.

CARD/RGI Term	Drug Class/Function	Identity (%)	Reference Length (%)
qacJ	SMR-family efflux; disinfecting agents/antiseptics	40.20	95.33
vanY (vanB cluster)	Glycopeptide resistance-associated target alteration	31.93	95.90

These database matches were interpreted in the context of their low sequence identity and do not, by themselves, establish a functional resistance phenotype.

## Data Availability

The raw data supporting the conclusions of this article will be made available by the authors on request.

## Supplementary Materials

The following supporting information can be downloaded at: https://www.mdpi.com/article/10.3390/nu18172796/s1, Figure S1: Gram-stained microscopic appearance of Lactiplantibacillus plantarum ATA-LPC98052 at increasing magnifications.; Figure S2. Exploratory MaAsLin2 longitudinal treatment × time coefficients for selected species-level taxa.; Figure S3. Genus-level MaAsLin2 longitudinal treatment × time interaction results.; Table S1. Top-ranked exploratory species-level treatment × time associations from the MaAsLin2 longitudinal analysis.; Table S2. Selected genus-level MaAsLin2 treatment × time interaction results.; Table S3. Threshold-dependent VFDB screening of Lactiplantibacillus plantarum ATA-LPC98052 using ABRicate.

## Abbreviations

The following abbreviations are used in this manuscript:

ALP	alkaline phosphatase
ALT	alanine aminotransferase
ARRIVE	Animal Research: Reporting of In Vivo Experiments
AST	aspartate aminotransferase
ASV	amplicon sequence variant
BDL	below detection limit
BHI	brain heart infusion
CFU	colony-forming unit
CRP	*C*-reactive protein
DADA2	Divisive Amplicon Denoising Algorithm 2
DMEM	Dulbecco’s Modified Eagle Medium
DMSO	dimethyl sulfoxide
DNA	deoxyribonucleic acid
dsDNA	double-stranded DNA
GRA	granulocytes
GRAS	Generally Recognized as Safe
H&E	hematoxylin and eosin
HCT	hematocrit
HGB	Hemoglobin
IBG	Izmir Biomedicine and Genome Center
ISO	International Organization for Standardization
LA	tissue not collected
LYM	lymphocytes
MIC	minimum inhibitory concentration
MON	monocytes
MTT	3-(4,5-dimethylthiazol-2-yl)-2,5-diphenyltetrazolium bromide
NA	not applicable
NC	negative control
NCBI	National Center for Biotechnology Information
ND	not determined
OECD	Organisation for Economic Co-operation and Development
PBS	phosphate-buffered saline
PC	positive control
PCoA	principal coordinates analysis
PCR	polymerase chain reaction
PLT	platelets
QIIME2	Quantitative Insights Into Microbial Ecology 2
QPS	Qualified Presumption of Safety
RB	recovery group
RBC	red blood cell count
rRNA	ribosomal RNA
SD	standard deviation
VBNC	viable but non-culturable
WBC	white blood cell count
WGS	whole-genome sequencing
