# Supplementary material for: Comprehensive In Vitro, Genomic, Microbiota, and Subacute Toxicological Safety Characterization of Lactiplantibacillus plantarum ATA-LPC98052
Source: Nutrients. 2026 Aug 26;18(17):2796. doi: 10.3390/nu18172796 (PMC13567210; doi:10.3390/nu18172796)
Supplement: Supplementary file 1 [file nutrients-18-02796-s001.zip › nutrients-4463680-supplementary.pdf]

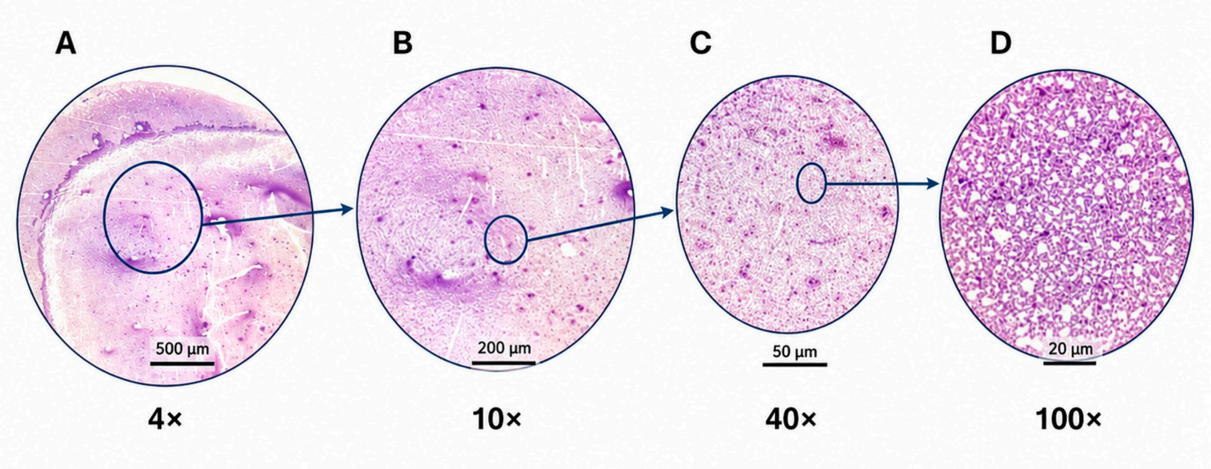

Supplementary Figure S1. Gram-stained microscopic appearance of *Lactiplantibacillus plantarum* ATA-LPC98052 at increasing magnifications. Representative images obtained at 4×, 10×, 40×, and 100× magnification illustrate the cellular distribution and morphology of the strain. The marked areas indicate the regions examined at progressively higher magnification.

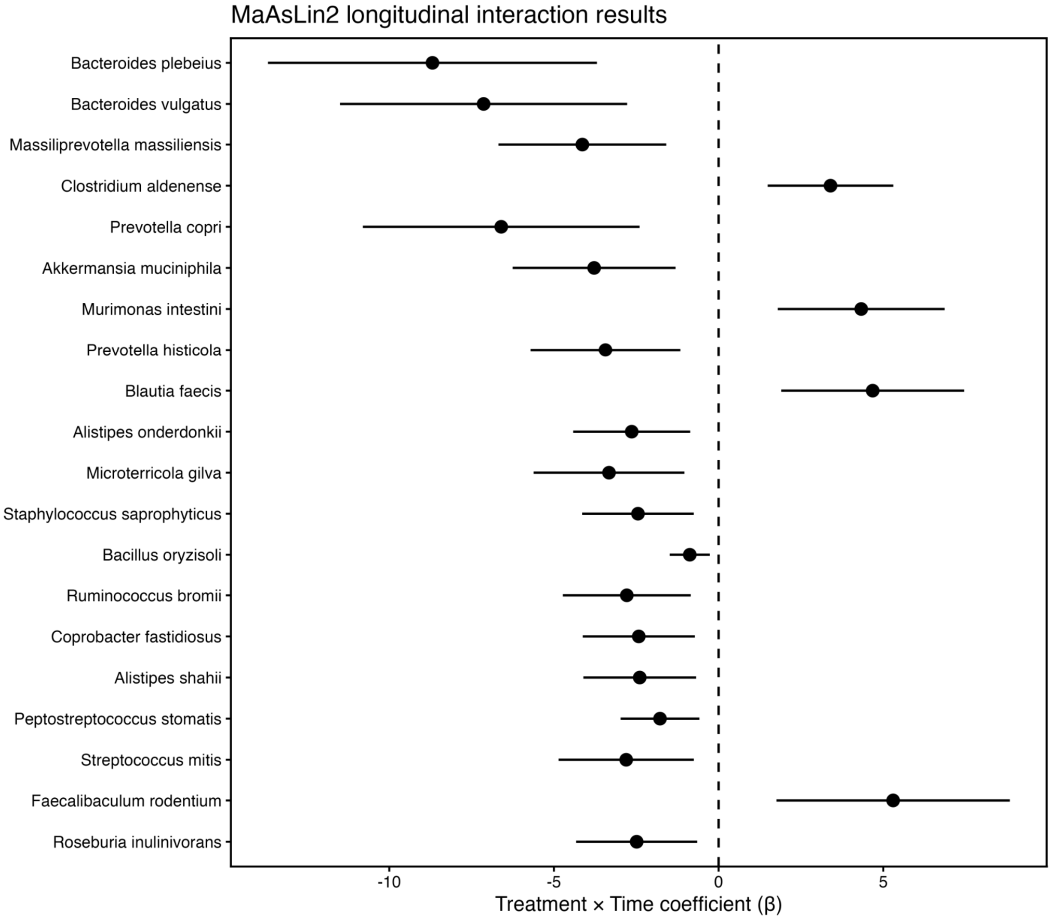

Supplementary Figure S2. Exploratory MaAsLin2 longitudinal treatment × time coefficients for selected species-level taxa. Points indicate model coefficients and horizontal lines indicate the corresponding uncertainty intervals. The dashed vertical line represents a coefficient of zero. No species-level association remained significant after false-discovery-rate correction at $q < 0.05$; therefore, the displayed signals should be interpreted as exploratory.

Supplementary Table S1. Top-ranked exploratory species-level treatment × time associations from the MaAsLin2 longitudinal analysis.

Taxon	Treatment × time coefficient	Raw p value	FDR q value
Bacteroides plebeius	-8.689	0.00278	0.1106
Bacteroides vulgatus	-7.136	0.00441	0.1251
Massiliprevotella massiliensis	-4.137	0.00468	0.1251
Clostridium aldenense	3.398	0.00582	0.1254
Prevotella copri	-6.601	0.00592	0.1254
Akkermansia muciniphila	-3.780	0.00716	0.1270
Murimonas intestini	4.330	0.00738	0.1270
Prevotella histicola	-3.435	0.00773	0.1270
Blautia faecis	4.679	0.00798	0.1270
Alistipes onderdonkii	-2.639	0.00865	0.1270

Note: A total of 1,573 species were evaluated. No species-level treatment × time association remained statistically significant after FDR correction at $q < 0.05$. The associations shown in Table S1 had q values < 0.25 and are presented only as exploratory signals; they should not be interpreted as confirmed treatment-specific differential abundance.

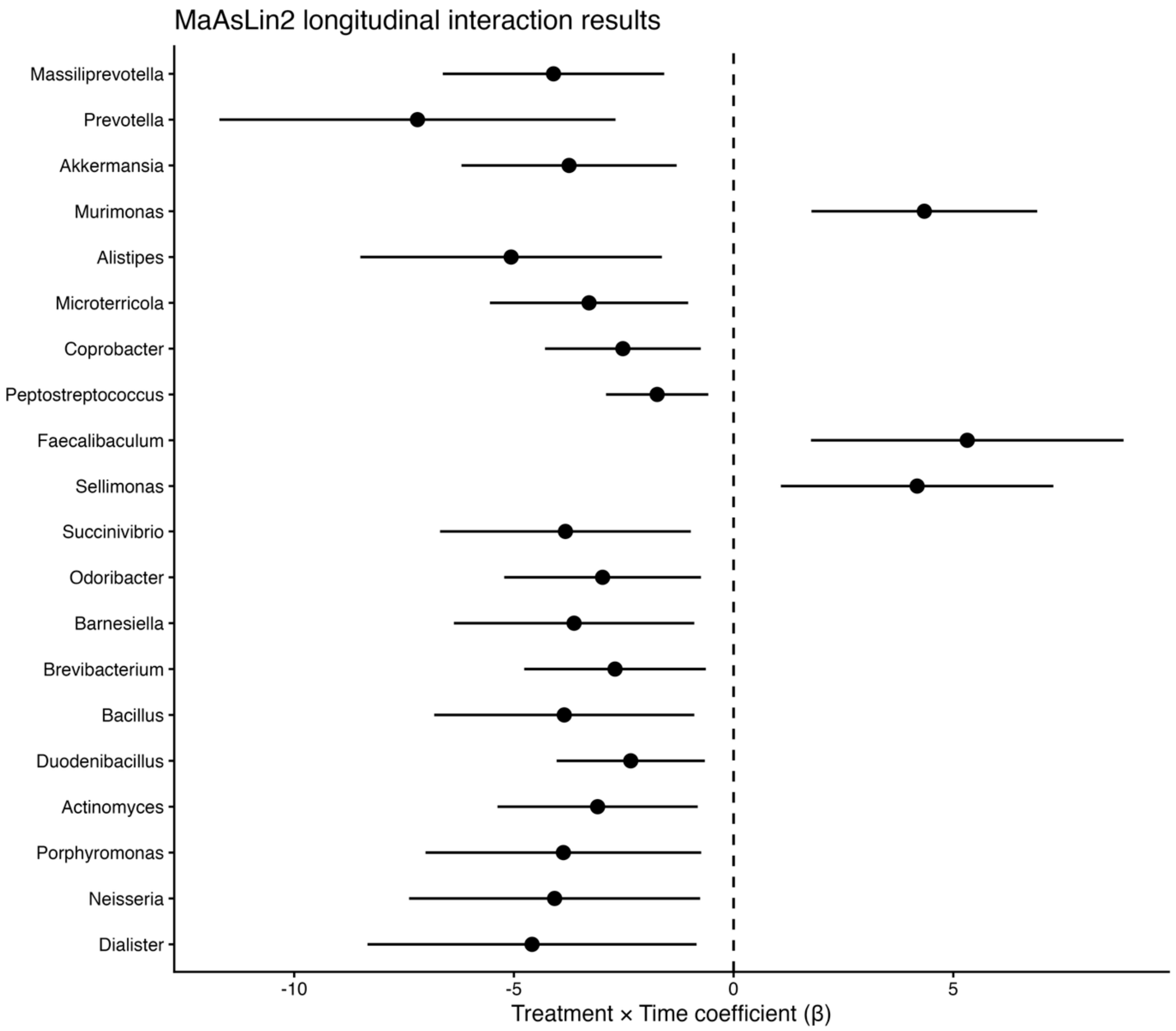

Supplementary Figure S3. Genus-level MaAsLin2 longitudinal treatment × time interaction results. The figure displays 20 top-ranked exploratory genera from the genus-level analysis. Points represent treatment × time coefficients (β), horizontal lines represent 95% confidence intervals, and the dashed vertical line indicates $\beta = 0$. Positive coefficients indicate a greater longitudinal increase in the probiotic group relative to the control group, whereas negative coefficients indicate a greater longitudinal decrease. No genus remained statistically significant after FDR correction at $q < 0.05$.

Supplementary Table S2. Selected genus-level MaAsLin2 treatment × time interaction results. The table includes the 20 top-ranked exploratory genera shown in Supplementary Figure S3 and four additional genera reported in the main text. A q-value < 0.05 was considered statistically significant after FDR correction; q < 0.25 was treated as exploratory.

Genus	Coefficient (β)	SE	Raw p-value	FDR q-value
Massiliprevotella	-4.099	1.286	0.0046	0.1411
Prevotella	-7.195	2.300	0.0053	0.1411
Akkermansia	-3.745	1.249	0.0071	0.1411
Murimonas	4.341	1.311	0.0079	0.1411
Alistipes	-5.064	1.751	0.0090	0.1411
Microterricola	-3.290	1.151	0.0097	0.1411
Coprobacter	-2.520	0.904	0.0114	0.1411
Peptostreptococcus	-1.740	0.594	0.0126	0.1411
Faecalibaculum	5.318	1.815	0.0150	0.1411
Sellimonas	4.177	1.582	0.0157	0.1411
Succinivibrio	-3.828	1.456	0.0161	0.1411
Odoribacter	-2.982	1.143	0.0168	0.1411
Barnesiella	-3.630	1.396	0.0171	0.1411
Brevibacterium	-2.699	1.054	0.0187	0.1411
Bacillus	-3.854	1.510	0.0190	0.1411
Duodenibacillus	-2.341	0.860	0.0215	0.1492
Actinomyces	-3.095	1.163	0.0238	0.1592
Porphyromonas	-3.874	1.601	0.0251	0.1620
Neisseria	-4.074	1.690	0.0257	0.1620
Dialister	-4.587	1.910	0.0262	0.1620
Bifidobacterium	-4.616	3.055	0.1464	0.3901
Citrobacter	-3.680	3.971	0.3651	0.6806
Lactobacillus	1.680	1.986	0.4078	0.7194
Romboutsia	-0.306	2.613	0.9079	0.9940

Note. Across all 593 genera tested, no treatment × time association met the FDR significance threshold of q < 0.05. The first 20 rows correspond to the top-ranked exploratory genera shown in Supplementary Figure S3. Lactobacillus, Bifidobacterium, Romboutsia, and Citrobacter did not show significant treatment × time interactions after FDR correction and did not meet the exploratory q < 0.25 threshold.

Supplementary Table S3. Threshold-dependent VFDB screening of Lactiplantibacillus plantarum ATA-LPC98052 using ABRicate.

Screening was performed with ABRicate v1.2.0 against the VFDB database (database version: 15 March 2026). Minimum sequence identity (ID) and gene coverage (COV) thresholds were tested from 50% to 90% in 10% increments.

Minimum ID (%)	Minimum COV (%)	No. of VFDB matches	Detected VFDB homologous matches
50	50	9	gndA, tufA, bsh, clpP, hasC, groEL, clpE, cpsI, wbtL
50	60	9	gndA, tufA, bsh, clpP, hasC, groEL, clpE, cpsI, wbtL
50	70	8	gndA, tufA, bsh, clpP, hasC, groEL, cpsI, wbtL
50	80	7	gndA, tufA, bsh, clpP, hasC, groEL, wbtL
50	90	4	gndA, tufA, clpP, groEL
60	50	9	gndA, tufA, bsh, clpP, hasC, groEL, clpE, cpsI, wbtL
60	60	9	gndA, tufA, bsh, clpP, hasC, groEL, clpE, cpsI, wbtL
60	70	8	gndA, tufA, bsh, clpP, hasC, groEL, cpsI, wbtL
60	80	7	gndA, tufA, bsh, clpP, hasC, groEL, wbtL
60	90	4	gndA, tufA, clpP, groEL
70	50	3	bsh, clpP, hasC
70	60	3	bsh, clpP, hasC
70	70	3	bsh, clpP, hasC
70	80	3	bsh, clpP, hasC
70	90	1	clpP
80	50	0	No hits
80	60	0	No hits
80	70	0	No hits
80	80	0	No hits
80	90	0	No hits
90	50	0	No hits
90	60	0	No hits
90	70	0	No hits
90	80	0	No hits
90	90	0	No hits

Note: Empty TSV outputs indicate that no VFDB match met the corresponding ID/COV thresholds. No hit annotated as Listeria adhesion protein (Lap) was detected under any tested threshold combination. No VFDB matches were detected at minimum identity thresholds of 80% or 90%.

Supplementary Table S3 (continued). Unique VFDB homologous matches detected at the lowest-stringency setting (ID 50% / COV 50%).

Gene	Sequence	Coordinates	Strand	Coverage (%)	Identity (%)	Accession	VFDB annotation / reference
gndA	NZ_JBEGGS010000001.1	190966–192361	+	96.38	64.17	WP_014907233	NADP-dependent phosphogluconate dehydrogenase; Capsule (VF0560) – Immune modulation (VFC0258); reference: Klebsiella pneumoniae subsp. pneumoniae NTUH-K2044
tufA	NZ_JBEGGS010000001.1	732168–733336	−	97.89	69.70	WP_003028672	Elongation factor Tu; EF-Tu (VF0460) – Adherence (VFC0001); reference: Francisella tularensis subsp. tularensis SCHU S4
bsh	NZ_JBEGGS010000002.1	103187–104069	−	89.98	72.12	NP_465591	Bile salt hydrolase; BSH (VF0350) – Stress survival (VFC0282); reference: Listeria monocytogenes EGD-e
clpP	NZ_JBEGGS010000003.1	195670–196228	+	93.47	70.71	NP_465991	ATP-dependent Clp protease proteolytic subunit; ClpP (VF0074) – Stress survival (VFC0282); reference: Listeria monocytogenes EGD-e
hasC	NZ_JBEGGS010000003.1	232778–233580	−	87.65	71.81	WP_010922799	UTP–glucose-1-phosphate uridylyltransferase HasC; Hyaluronic acid capsule (VF0244) – Immune modulation (VFC0258); reference: Streptococcus pyogenes M1 GAS
groEL	NZ_JBEGGS010000003.1	258446–260023	−	95.64	65.96	WP_003435012	Chaperonin GroEL; GroEL (VF0594) – Adherence (VFC0001); reference: Clostridium difficile 630
clpE	NZ_JBEGGS010000010.1	13085–14532	+	65.70	66.60	NP_464522	ATP-dependent protease; ClpE (VF0073) – Stress survival (VFC0282); reference: Listeria monocytogenes EGD-e
cpsI	NZ_JBEGGS010000010.1	59548–60397	−	73.86	66.82	WP_002376666	UDP-galactopyranose mutase; Capsule (VF0361) – Immune modulation (VFC0258); reference: Enterococcus faecalis V583
wbtL	NZ_JBEGGS010000010.1	80564–81345	−	87.46	64.71	WP_003018140	Glucose-1-phosphate thymidylyltransferase; LPS (VF0542) – Immune modulation (VFC0258); reference: Francisella tularensis subsp. tularensis SCHU S4

The entries above are VFDB database homologous matches and should not, by themselves, be interpreted as evidence of functional pathogenic virulence.
